# Molecular exaptation by the integrin αI domain

**DOI:** 10.1126/sciadv.adx9567

**Published:** 2025-09-10

**Authors:** Jeremy A. Hollis, Matthew C. Chan, Harmit S. Malik, Melody G. Campbell

**Affiliations:** ^1^ Division of Basic Sciences, Fred Hutchinson Cancer Center, Seattle, WA 98109, USA.; ^2^ Graduate Program in Molecular and Cellular Biology, University of Washington, Seattle, WA 98195, USA.; ^3^ Howard Hughes Medical Institute, Seattle, WA 98109, USA.

## Abstract

Integrins bind ligands between their alpha (α) and beta (β) subunits and transmit signals through conformational changes. Early in chordate evolution, some α subunits acquired an “inserted” (I) domain that expanded integrin’s ligand-binding repertoire but obstructed the ancestral ligand pocket, seemingly blocking conventional integrin activation. Here, we compare cryo–electron microscopy structures of apo and ligand-bound states of the I domain–containing αEβ_7_ integrin and the I domain–lacking α_4_β_7_ integrin to illuminate how the I domain intrinsically mimics an extrinsic ligand to preserve integrin function. We trace the I domain’s evolutionary origin to an ancestral collagen-collagen interaction domain, identifying an ancient molecular exaptation that facilitated integrin activation immediately upon I domain insertion. Our analyses reveal the evolutionary and biochemical basis of expanded cellular communication in vertebrates.

## Teaser

An ancient protein domain insertion seamlessly preserved conformational signaling while providing a novel surface for adaptation.

## INTRODUCTION

**Fig. 1.**
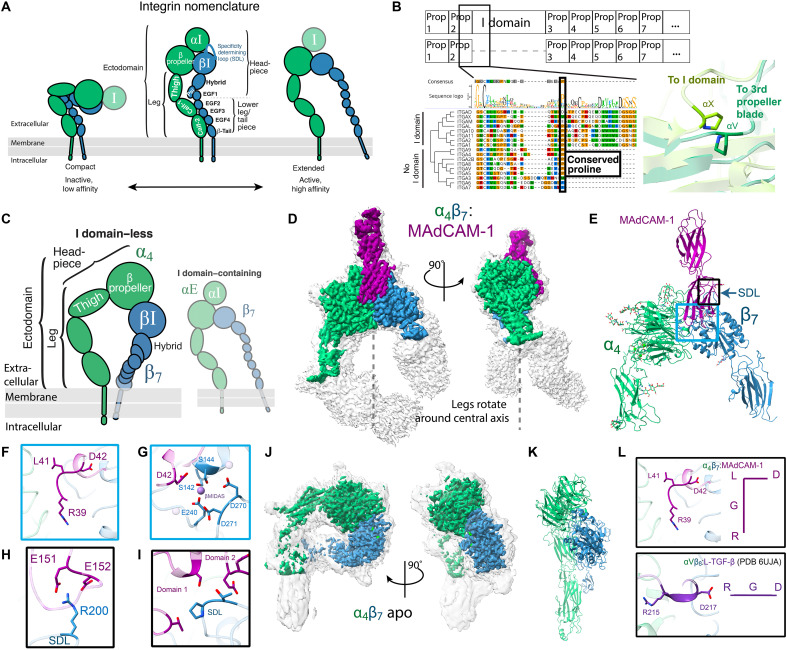
Integrin α_4_β_7_ adopts distinct conformations. (**A**) A schematic of integrin organization. Conformations range from a compact, inactive state with low ligand affinity (left) to an extended-open, active state with high ligand affinity (right). Hypothesized I domain locations are semitransparent. (**B**) A human integrin α subunit alignment shows the I domain inserted in the ancestral integrin gene immediately following a conserved proline at the end of the second β-propeller blade. Structural overlays of the I domain–less integrin αV [dark green; Protein Data Bank (PDB) 1L5G (*105*)] and I domain–containing αX [lime green; PDB 4NEH (*10*)] with their respective prolines displayed. Prop, propeller. (**C**) A schematic highlighting the focus of this figure on the I domain–lacking integrin α_4_β_7_. (**D**) Cryo-EM structure of the integrin α_4_β_7_ ectodomain (green or blue) bound to the mucosal addressin cell adhesion molecule–1 (MAdCAM-1) ectodomain (purple). The low-threshold, unsharpened global map is shown in gray, and the high-threshold, sharpened local map is overlaid in color. The legs of α_4_ and β_7_ rotate around a vertical axis. (**E**) Atomic model of the α_4_β_7_:MAdCAM-1 complex. Blue box indicates regions of focus in (F) and (G), and black box indicates region of focus in (H) and (I). (**F**) MAdCAM-1 uses an RGLD motif instead of the canonical tripeptide RGD to bridge the α_4_ and β_7_ subunits. The RGLD residues span the long axis of the cleft formed between α_4_ and β_7_. (**G**) MAdCAM-1 D42 completes the β_7_ metal ion–dependent adhesion site (βMIDAS) ion coordination sphere, here modeled as Mn^2+^. (**H**) R200 in the β_7_ specificity-determining loop (SDL) forms essential salt bridges with a flexible loop in MAdCAM-1. (**I**) Stabilizing contacts intersect between the first and second domains of MAdCAM-1 with the β_7_ SDL. (**J**) The cryo-EM structure and (**K**) rigid-fit model of the apo-compact α_4_β_7_. (**L**) The RGLD motif in MAdCAM-1 adopts a right angle, in contrast to the canonical linear RGD motif of αVβ_8_-bound latent transforming growth factor–β (L-TGF-β) (*106*).

Protein evolution often proceeds via small incremental mutational steps. In contrast, protein domain acquisition can spur marked novelty, sometimes at the cost of ancestral functions (*1*). One such marked domain acquisition occurred early in chordate evolution in the integrin family of cell surface receptors, which mediate signaling across the cell membrane that is critical for various biological processes, ranging from embryogenesis to T cell activation. Integrins are heterodimeric proteins composed of a single alpha (α) and beta (β) subunit (Fig. 1A). Integrins canonically exist in a conformational spectrum from a closed, compact state to an open, extended state, with the closed state considered inactive and the open state considered the major signaling state (Fig. 1A). The human genome encodes 8 β- and 18 α-integrin subunits, 9 of which include a 200 amino acid–derived “inserted” (I) von Willebrand factor type-A (vWFA) domain (or I domain) (*2*–*6*). Integrin α subunits encode seven blades of a β-propeller fold, with the I domain found between the second and third propeller blades (Fig. 1B) (*7*).

Integrins lacking I domains bind ligands at an interface between α and β subunits. Ligand binding is also directly linked to the conformational changes required to signal integrin activation by a canonical RGD tripeptide present in many extrinsic ligands (*8*). However, the insertion of the I domain sterically occluded this ligand-binding interface, potentially disrupting both ligand binding and the allostery required for integrin activation. Prior studies showed that the I domain acquired ligand-binding functions, enabling I domain–containing integrins to bind a wider array of ligand moieties and motifs, including collagens, complement components, and other extracellular matrix proteins (*9*). Moreover, structural characterization of isolated I domains revealed a conformational change in their C-terminal helix that inserts a conserved glutamate into the ancestral ligand-binding pocket of integrin. This shifted glutamate may structurally and functionally substitute for the RGD motif, coordinating a cation essential for integrin extension (*10*). However, the conformational states of an I domain–containing integrin have never been observed with the required structural detail to resolve ligand-binding and integrin activation features. As a result, the mechanism by which domain-triggered integrin activation occurs is still not well understood. Given that half of human α-integrins contain I domains—including most with critical immune functions—resolving the molecular details of this evolutionary insertion would provide vital insight into a wide array of vertebrate signaling processes and reveal fundamental principles of how protein domains are successfully co-opted.

Here, we use cryo–electron microscopy (cryo-EM), computational heterogeneity, biochemical, and evolutionary analyses to dissect the evolutionary and mechanistic basis of this pivotal protein domain insertion. We directly compare the activation networks of two integrin heterodimers involving β_7_, a β-integrin subunit capable of interacting with both types of α-integrin subunits (Fig. 1C). The β_7_ integrin family, encompassing α_4_β_7_ [Lymphocyte Peyer’s patch adhesion molecule-1 (LPAM-1)] and αEβ_7_ (CD103), performs critical roles in facilitating immune cell homing, activation, and retention at sensitive immune barriers (*11*, *12*). Thus, in addition to revealing unprecedented insights into the fundamental biochemical and evolutionary origins of allostery underlying integrin activation, our structures of the α_4_β_7_ and αEβ_7_ integrins have substantial clinical value (*13*–*15*).

## RESULTS

### Integrin α_4_β_7_ adopts distinct conformations

We first determined a structure of the non-I α_4_β_7_ integrin ectodomain bound to its cognate ligand mucosal addressin cell adhesion molecule–1 (MAdCAM-1) (Fig. 1, D and E, figs. S1 and S13, and table S1). This interaction is critical for leukocyte migration to the gut (*16*), yet no previous structure of the complex has been reported. We find MAdCAM-1 fits snugly within the groove between α_4_ and β_7_, forming interactions with both integrin subunits (fig. S2A). Contacts primarily occur on the first immunoglobulin (Ig)–like MAdCAM-1 domain, between the MAdCAM-1 FG strands and α_4_ propeller and between the MAdCAM-1 CD loop and the β_7_ βI domain (fig. S2, A and B). MAdCAM-1 centrally bridges the α_4_ and β_7_ subunits with an RGLD motif; R39 and L41 span the long axis of the integrin groove, filling the space between α_4_ and β_7_ (Fig. 1F). The D42 residue provides the essential ion coordination activity within this motif, leading to the open integrin conformation characteristic of an active heterodimer (Fig. 1G). These central contacts are further stabilized by the MAdCAM-1 DE β ribbon loop in the Ig-like domain 2 (D2) contacting the β_7_ specificity-determining loop (SDL) via mutationally intolerant (*17*) hydrogen bonds (Fig. 1H), supported by an additional interface between MAdCAM-1 D1, D2, and the β_7_ SDL (Fig. 1I).

Integrins have been proposed to unfold similarly to a switchblade, as they transition from their compact to open states (*18*, *19*). Accordingly, we observe a high degree of β_7_ leg flexibility in the α_4_β_7_:MAdCAM-1 structure (movie S1) within the open headpiece conformation. However, by resolving the previously (*20*) undescribed lower leg region of the α_4_ subunit encompassing the calf-1 and calf-2 domains, we found that the open α_4_β_7_ conformation has a slight rotation around a vertical axis (Fig. 1D). To accommodate the different orientations of the headpiece relative to the legs between the open and closed α_4_β_7_, we propose that α_4_β_7_ must “untwist” open rather than “unfold” such as in the classic switchblade model. We determined an apo α_4_β_7_–clasped structure under high-calcium buffer conditions previously shown to favor the inactive conformational state (Fig. 1, J and K; figs. S3, A to E, and S13; and table S1) (*21*). In this apo structure, α_4_β_7_ defines a noncanonical bent conformation. The headpiece is skewed, and the β_7_ headpiece region sits below the α_4_ propeller, mediated by contacts occurring between α_4_ calf-1 and the plexins, semaphorins, and integrins (PSI)/epidermal growth factor 1 (EGF1) region of β_7_ (fig. S3F). We propose that the simplest means for α_4_β_7_ to transition from a bent to an extended state would be by twisting about the lower leg region. Three-dimensional (3D) flexibility analysis of the α_4_β_7_:MAdCAM-1 complex supports this hypothesis (movie S1), revealing a continuous ensemble of conformational twisting. Given our structural data, this “twisted” compact orientation might also explain how α_4_β_7_ binds MAdCAM-1 perpendicular to the orientation at which α_5_β_1_ binds fibronectin (fig. S3F) (*22*, *23*), despite both integrins having a similar angle of ligand approach or occlusion.

In addition to distinct conformations, we find several other idiosyncrasies that confer specificity to the α_4_β_7_:MAdCAM-1 complex. Our open structure reveals that MAdCAM-1 undergoes a conformational shift upon binding integrin. In contrast to existing crystal structures of MAdCAM-1 alone (*24*, *25*), we find that the MAdCAM-1 D β strand runs antiparallel to the E strand in D1 when bound to α_4_β_7_ (fig. S2C). Furthermore, in contrast to the well-characterized linear RGD motif within αV integrin ligands (*26*), we see that the RGLD present in MAdCAM-1 is at a right angle to fit within the exceptionally deep perpendicular groove unique to α_4_β_7_ (Fig. 1L), emphasizing the plasticity of this canonical motif. Prior mutational studies (*17*, *27*, *28*) of integrin and MAdCAM-1 have independently shown the importance of β_7_ ion coordination sites and each MAdCAM-1 domain, including a proposed integrin-binding loop in MAdCAM-1 D1 and acidic patch in D2. Our structural data allow us to unite these studies into a cohesive stepwise model of specific ligand binding (*27*). We propose that integrin initially surveys immune sites via the SDL/D2 interaction distal to the cell surface when integrin is bent and then favors cells slowing to roll via tighter dynamic interactions influencing the β_7_ synergistic metal ion–binding site (SyMBS), eventually adhering firmly to the endothelial cell layer via MAdCAM-1 slotting specifically into the α_4_β_7_ groove, triggering integrin untwisting (fig. S2D). We propose that the “untwisting” of α_4_β_7_ we observe in our structural data represents the connection between the intermediate transition state observed in prior low-resolution structural studies of α_4_β_7_ (*29*) and the open state from our study, collectively revealing the pathway toward integrin activation. While our model plausibly unifies our current understanding of α_4_β_7_-mediated adhesion, future studies of high-resolution intermediate states will clarify the timing and sequence of these molecular events. Together, our α_4_β_7_ structures represent two unique integrin conformations and reveal specificity mechanisms governing the α_4_β_7_:MAdCAM-1 interaction.

### The inactive αEβ_7_ I domain is unexpectedly dynamic

**Fig. 2.**
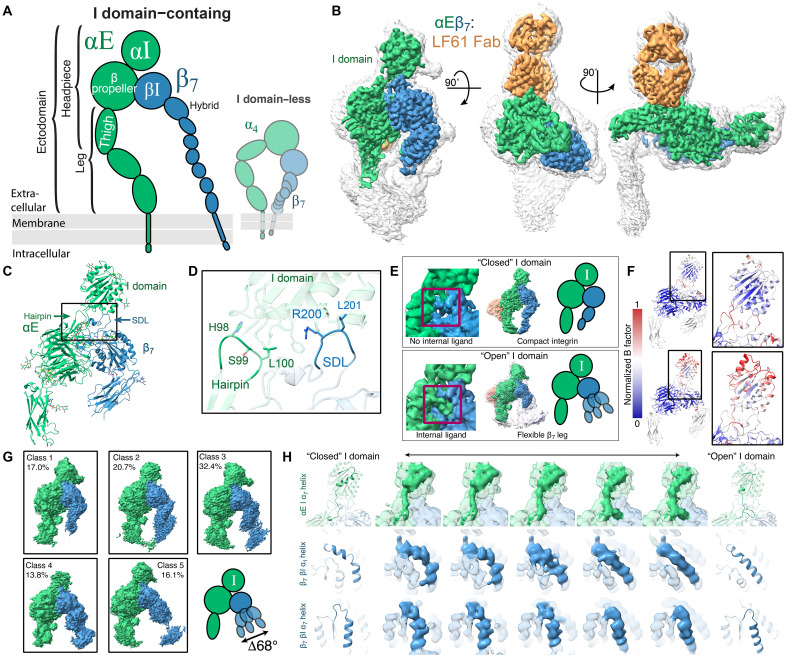
The half-bent αEβ_7_-inactive structure is dynamic. (**A**) A schematic highlighting the focus of this figure on the I domain–containing integrin αEβ_7_. (**B**) Three views of the αEβ_7_:LF61 Fab complex map. αE (green), β_7_ (blue), and LF61 (orange). The colored, sharpened, high-threshold map is shown over the unsharpened, grayscale, low-threshold map. (**C**) The molecular model of the compact αEβ_7_ headpiece. All ions are modeled as Ca^2+^. Black box indicates region of focus in (D) and (E). (**D**) The αE I domain is stabilized by supports from a β-hairpin on the αE propeller domain and the β_7_ SDL. (**E**) αEβ_7_ assumed two broad states. In the closed I domain state (left), the C-terminal region of the α_7_ helix, termed the internal ligand, does not engage with the β_7_ subunit, and the β_7_ leg is closed inward. In the open I domain state (right), the internal ligand engages β_7_, leading to conformational heterogeneity in the β_7_ leg. For both structures, the unsharpened map from a nonuniform refinement is shown in color; for the open I domain, representative structures from 3D classification are shown overlaid with low opacity, and a schematic is shown to the right. Red boxes indicate the internal ligand region. (**F**) B factor analysis, a quantitative representation of protein motion via thermal energy, shows that the I domain becomes broadly more dynamic in the open conformation. (**G**) 3D classification of five open I domain classes suggests a continuous range of β_7_ hybrid domain states upon internal ligand engagement. (**H**) Representative intermediates from 3D variability analyses show that the engagement of the I domain internal ligand is directly linked to the opening of the β_7_ βI domain, leading to subsequent hybrid domain swingout.

Next, we focused on the structural analysis of the I domain–containing αEβ_7_ integrin. Negative-stain electron microscopy (nsEM) of the purified αEβ_7_ ectodomain under several cation conditions (fig. S4) revealed both closed-state and open-state conformations. To gain more detailed insight into the compact inactive state of the I domain (Fig. 2A), we performed cryo-EM on the αEβ_7_ ectodomain under nonactivating buffer conditions. To facilitate structural analysis, we bound this integrin to LF61, an αE antibody fragment that does not influence ligand binding (*30*). Unexpectedly, 3D analysis revealed two distinct populations of particles. The first, which we had expected to see, was the compact conformation, which we determined to a global resolution of 2.9 Å (Fig. 2B and figs. S5, S6, and S13). The intrinsic dynamic variability of the leg results in the overall resolution of the map being quantitatively anisotropic (figs. S5 and S6). Nevertheless, most of the headpiece region was rigid, enabling us to confidently model the αEβ_7_ headpiece region (Fig. 2C and table S1) just as we did for α_4_β_7_. The I domain is positioned above the heterodimer interface, which would completely sterically occlude the ancestral RGD pocket of I domain–lacking integrins. We found that the αE I domain makes additional contacts with the β_7_ SDL and a β-hairpin on the αE β propeller. These supports form a “collar” around the αE I domain (Fig. 2D), contributing to the stability necessary for this integrin to perform its biological function in maintaining persistent epithelial contacts (*31*–*35*). αE I domain contacts are exceptionally extensive; the αE I domain has a 940-Å^2^ contact area with the rest of the integrin heterodimer, whereas the αM I domain from Protein Data Bank (PDB) 7P2D (*36*) has only a 493.4-Å^2^ contact area with the rest of the I domain–containing αMβ_2_ integrin.

The I domain contains an internal ligand motif (*37*) proposed to coordinate the cation site β metal ion–dependent adhesion site (βMIDAS) in the β subunit via a conserved acidic glutamate. The second population of particles not only showed appreciable heterogeneity in the degree of headpiece opening (Fig. 2E, fig. S6, and movie S2) but also revealed engagement of the internal ligand. This was unexpected given the high Ca^2+^ levels in this buffer, which have been shown to favor an inactive conformation for many other well-studied integrins (*21*). Consistent with previous analyses of isolated I domain ectodomains (*10*), we found that extension of the I domain’s C-terminal α_7_ helix is a prerequisite for internal ligand binding and βMIDAS coordination. This helix in our structure of the internally liganded αEβ_7_ occupied the “open” conformation previously described in crystal structures of other I domains (*38*). However, in contrast to the metastable state occupied by αXβ_2_ (*10*), we found that the αEβ_7_ headpiece becomes broadly dynamic upon internal ligand binding (Fig. 2, E and F). 3D classification analysis suggested that the β_7_ hybrid domain adopts a continuous range of motion in this internally liganded state (Fig. 2G). Next, using 3D variability analysis (*39*), we mapped how the dynamic behavior of the I domain helix-7 is directly linked to the deformation of key βI domain helices in a stepwise manner (Fig. 2H). These dynamics are unique to integrins containing the I domain; integrins lacking I domains cannot sample these states in the absence of ligand, as evidenced by our α_4_β_7_ apo structure.

### Ligand binding stabilizes the open αEβ_7_ I domain

**Fig. 3.**
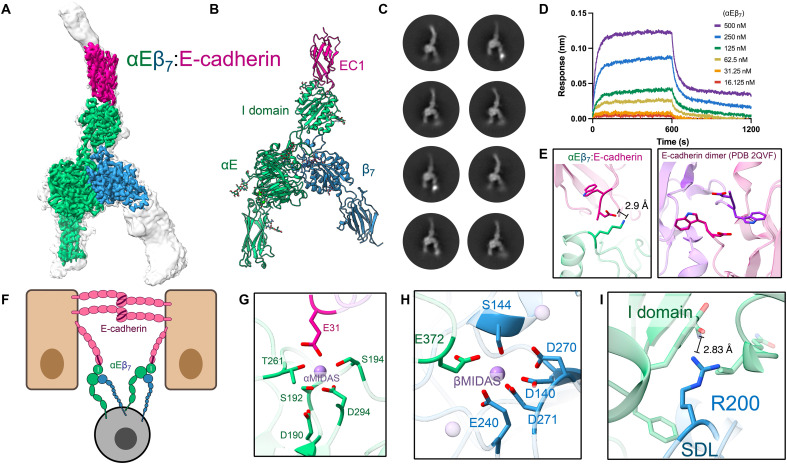
The molecular interface between integrin αEβ_7_ and E-cadherin. (**A**) Cryo-EM structure of the integrin αEβ_7_ ectodomain (green or blue) bound to the first two EC domains of ligand E-cadherin (pink). The low-threshold, unsharpened global refinement map is shown in gray, and overlaid is the high-threshold, sharpened local refinement map in color. The open integrin legs suggest an active conformation. (**B**) The atomic model of the αEβ_7_:E-cadherin complex. Top black box indicates region of focus in (D). (**C**) Representative 2D class averages of αEβ_7_ bound to EC12 suggest that αEβ_7_ binds exclusively to E-cadherin monomers. (**D**) BLI data suggest that E-cadherin and αEβ_7_ form a high-affinity, stable interaction. (**E**) A salt bridge between the αE I domain and the first residue in the mature E-cadherin prevents the tryptophan strand-swap engagement essential for adherens junction formation (*64*). (**F**) A competitive inhibition model for T cell tissue residence. αEβ_7_ (CD103) outcompetes E-cadherin trans-dimerization, permitting stable epithelial tissue residence by CD103^+^ cells. (**G**) The αE I domain (green) αMIDAS’ ion (purple) coordination sphere is completed by E-cadherin (pink) E31. (**H**) The β_7_ βMIDAS coordination sphere is completed in the ligand-bound αEβ_7_ via the internal ligand E372. (**I**) The β_7_ R200 residue stabilizes the αE I domain with hydrophobic and charge-based interactions.

To understand how the I domain internal ligand movement is connected to external ligand binding, we next determined a cryo-EM structure of the αEβ_7_ ectodomain bound to its primary ligand E-cadherin (Fig. 3A and figs. S7 and S13) (*40*). At a global resolution of 3.4 Å, the ligand-binding interface was well resolved; we modeled the integrin headpiece region and E-cadherin EC1 (Fig. 3B). Our structure resolves the previous uncertainty about which E-cadherin domains are necessary for integrin binding (*41*). We find E-cadherin EC1 exclusively binds to the distal surface of the αE I domain, using a short E-cadherin construct (EC12) (Fig. 3A) (*42*). E-cadherin occupies physiological monomeric and dimeric states, with trans-dimerization between adjacent epithelial cells serving as the essential building block of epithelial tissue via higher-order structures termed adherens junctions (*43*). Despite this, we observed that E-cadherin is only present in a monomeric state when bound to αEβ_7_ (Fig. 3C). The αEβ_7_:E-cadherin molecular interface is primarily mediated by a central hydrophobic “lock and key” pocket surrounded by a network of electrostatic bonds (fig. S8, A and B, and data S2) (*44*). This interface is further stabilized by the N303 glycosylation on the αE I domain, which makes direct contact with E-cadherin (fig. S8C). The αEβ_7_:E-cadherin complex is highly stable, with a dissociation constant (*K*_d_) of 51 ± 7.9 nM measured using biolayer interferometry (BLI) (Fig. 3D). This structure pinpoints molecular interactions essential for sustained tissue residence (T_rm_) that are effectuated by αEβ_7_ (*31*–*34*). We found that the αE I domain forms a salt bridge with residue 1 of the mature E-cadherin molecule (Fig. 3E), effectively outcompeting adherens junctions by preventing the “strand-swap” step necessary for E-cadherin’s trans-dimerization (*45*, *46*) due to E-cadherin’s higher affinity to αEβ_7_ than to itself (Fig. 3F).

Integrin I domains contain a cation-binding site (αMIDAS) coordinated by a conserved DXSXS motif. The αE I domain αMIDAS is ion occupied in our structure, and the ion coordination complex is completed by E-cadherin E31 (Fig. 3G). Thus, consistent with other integrin I domains (*47*–*49*), αMIDAS coordination also leads to an open αE I domain, with its C-terminal helix assuming an extended conformation (fig. S8D). Despite this marked shift, the angle of the I domain relative to the apo structure remains almost entirely unchanged upon ligand binding (fig. S8E). These findings are consistent with our evidence from the compact structure that the αE I domain is exceptionally stable compared to other well-studied integrins. The ligand-bound αEβ_7_ integrin assumes an open headpiece conformation. However, 3D flexibility analysis of the dataset shows that the β_7_ leg still maintains a notable, yet restricted, degree of free movement (27° β_7_ hybrid movement compared to 68° in the apo-open structure; movie S3). This intrinsic flexibility allows integrin to remain ligand bound and resident even while permitting dynamic cell movement and changes in membrane morphology.

In contrast to the unliganded structure, the I domain internal ligand is consistently engaged with β_7_ when bound to E-cadherin. In this open conformation, the I domain interactions shift markedly. For example, instead of contacting residues E372 to D377 of the αE propeller hairpin in the closed structure, the collar contacts I domain residues Y366 to I369 (fig. S8F). This shift allows the αE I domain to maintain its rigidity in both inactive and active states and reinforces the helix-7 allosteric structural platform that links ligand binding to integrin activation. Our structures provide an unprecedented opportunity to visualize and compare conformational changes experienced by β_7_ upon binding of an external ligand in the α_4_β_7_ non–I domain configuration or by an internal ligand in an I domain–containing αEβ_7_ heterodimer. We find unambiguous support for the structural mimicry of external ligand–mediated integrin activation by the intrinsic I domain at key interaction sites. αE E372 in the αEβ_7_ heterodimer coordinates the same βMIDAS ion as MAdCAM-1 D42 within the α_4_β_7_:MAdCAM-1 complex (Fig. 3H). Moreover, the β_7_ SDL uses the identical R200 residue to support MAdCAM-1 as it does the αE I domain (Fig. 3I). This leads to the same open conformation and similar leg flexibility in α_4_β_7_ (35° β_7_ hybrid movement) as seen in internally liganded αEβ_7_ (27° β_7_ hybrid movement). The root mean square deviation (RMSD) of the β_7_ βI domain in ligand-bound α_4_β_7_ versus αEβ_7_ is 0.6 Å, indicating that β_7_ ultimately undergoes nearly identical conformational behavior in the context of either α_4_ or αE when activated. Thus, despite the presence of an entirely new domain, this suggests that the core activation mechanism has been preserved between the ancestral and derived integrin states. Collectively, our β_7_ structures reveal an intimate incorporation of the I domain with the ancestral integrin machinery.

### The integrin I domain was derived from collagen

Our findings reveal that the evolutionarily ancient I domain insertion expanded the ligand-binding repertoire of integrin proteins without perturbing the ancestral intricate conformational changes required for activation. We speculated that, right from its inception, the I domain’s ancestor might already have encoded all the necessary machinery to facilitate the allosteric communication between ligand binding and integrin activation seen in our structures, including the dynamic helix-7. To test this hypothesis, we aligned a comprehensive selection of integrin I domains across Olfactores (tunicates and vertebrates) to find the common ancestor of all I domains. We used this alignment to generate a hidden Markov model (HMM) of the protein domain encompassing the I domain and internal ligand, which we used to query the genomes of human, coelacanth, echinoderms, and Cephalochordata, a sister group to Olfactores. This strategy enabled us to identify homology to the presumed I domain ancestor rather than the multiple other vWFA domains encoded by animal genomes (fig. S9A) (*50*).

**Fig. 4.**
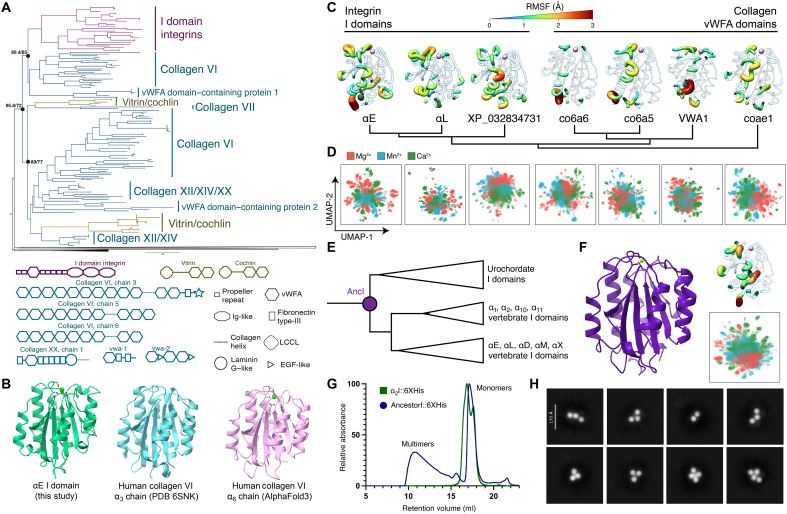
The integrin I domain was co-opted from an ancient collagen gene. (**A**) A phylogenetic tree of the vWF-like domains of the top hits from our HMM search was generated using IQ-TREE. The integrin I domain is most closely related to vWF-like domains from collagen VI proteins. (**B**) Atomic models of the closed I domain (this study), a human collagen VI α_3_ (PDB 6SNK) vWFA-like domain, and an AlphaFold3 prediction of a vWFA-like domain of human collagen VI α_6_ chain (UniProt C3YQB2) show high visual structural similarities. (**C**) MD simulations of representative collagen or integrin vWFA domains bound with Mg^2+^ in the MIDAS site suggest that characteristic dynamics were present in the common integrin I domain ancestor. The individual residue flexibility [i.e., root mean square fluctuations (RMSF)] is projected on the 3D structure and colored from 0 (blue) to >3 (red). Regions of fluctuations greater than 1.0 Å are highlighted. Model details are provided in table S2. (**D**) UMAP representations of conformational landscape of the representative collagen or integrin vWFA domains in (C). A total of 60,000 simulated conformations are projected on the UMAP and colored by the bound ion in the MIDAS site. (**E**) Phylogenetic tree schematic of sequences used for sequence reconstruction of the ancestral integrin I domain. (**F**) An AlphaFold3 structural prediction of the ancestral integrin I domain (left) and RMSF calculations and UMAP projection from MD simulations (right). RMSF is colored as in (C). UMAP is colored as in (D). (**G**) Size exclusion chromatography traces of recombinant 6xHis-tagged I domains. The trace for the extant collagen-binding α_2_ I domain is in green, and the trace for the ancestral reconstruction construct is in blue. (**H**) Representative negative-stain class averages of ancestral I domain multimers.

Our analyses reveal that the most likely ancestors of the α-integrin vWFA-like I domains are vWFA domains from extracellular matrix proteins, specifically those of the collagen VI class and its relatives (Fig. 4A). Outgroup collagen proteins share high structural homology with the αE I domain (Fig. 4B). To understand the origin of the I domain’s dynamic motion, we performed molecular dynamic (MD) simulations on 10 vWFA domains, which include outgroup collagen domains and extant integrin I domains, from published experimental structures and high-confidence structural predictions (fig. S10A and table S2). We found that the structural dynamics are concentrated in homologous regions among these proteins. Moreover, in addition to the overall vWFA fold, the dynamic motions of helix-7 are also conserved among these collagen and integrin I domains, supporting a common ancestry of these proteins (Fig. 4C and fig. S10A). Integrin I domain behavior is ion context dependent; the presence of calcium, magnesium, or manganese cations differentially influences physiological activation states via structural ion coordination sites (*51*). To test whether outgroup domains display similar behaviors, we performed these simulations in each ion condition and projected the conformational landscape of each domain as a uniform manifold approximation and projection (UMAP). We found that all collagen and integrin I domains sampled unique ion-specific conformations, suggesting that the differential behaviors of the integrin I domain under varying ions are an ancestral trait (Fig. 4D and fig. S10B).

Collagen proteins, especially collagen VI, commonly form oligomeric structures within or between collagen types, which are essential for their homointeractions (*52*). To directly test whether the ancestral I domain behaved similarly, we used Phylogenetic Analysis Using Maximum Likelihood (PAML) (*53*) to computationally reconstruct the common ancestor of all integrin I domains using the I domain alignment and phylogeny (Fig. 4E and fig. S9B). AlphaFold2 (*54*) modeling suggested that this ancestor structurally resembles extant integrin I domains, and molecular simulations showed that this ancestral domain contains the same dynamic behavior as extant integrin I domains (Fig. 4F and fig. S10). We expressed and purified this ancestral reconstruction and performed size exclusion chromatography to reveal that it forms multiorder oligomers; this contrasts with the extant α_2_ I domain, which only ran as a monomer (Fig. 4G). Negative-stain analysis revealed oligomers highly reminiscent of those formed by extant collagen VI (Fig. 4H). On the basis of our analyses, we infer that the ancestral I domain could bind collagen immediately upon acquisition. Consistent with this inference, extant integrin I domains across the chordate phylogeny bind collagens of varying types, including collagen VI (*55*). Our ancestral multimer data are phylogenetically most consistent with an ancient avidity-based mode of collagen binding, similar to that used by an extant tunicate I domain (*56*). This ancestral mechanism is independent of the canonical collagen helical GFOGER motif more recently acquired by vertebrate integrin I domains (*57*). Furthermore, the ion-coordinating MIDAS, which is completely intact in the ancestral sequence, performs critical functions in extant collagens that contain it (*58*, *59*), just as it does in integrin I domains. Our findings suggest that ion-dependent behavior was retained from the I domain’s initial co-option.

### The allosteric internal ligand was a de novo molecular exaptation

**Fig. 5.**
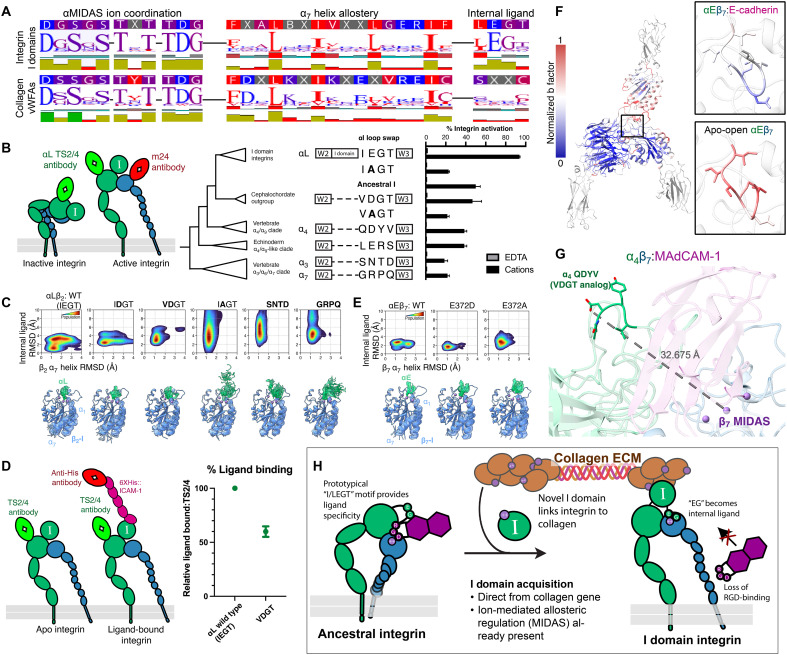
The internal ligand was exapted for integrin activation. (**A**) Logo plots of alignments of either representative I domains (top) or domains from outgroup collagens (bottom) show that important sequence features were likely present in the integrin I domain common ancestor except the βMIDAS ion-coordinating glutamate (right). Hydrophobicity trace is directly below; the bottom trace displays sequence conservation. A 25% consensus identity is displayed above. (**B**) Left: A schematic of the flow cytometry approach used to measure integrin activation in loop-swap experiments within an αLβ_2_ backbone. TS2/4 is a conformation-agnostic αL antibody, while m24 recognizes the active β_2_ subunit. Right: The internal ligand IEGT within the αLβ_2_ backbone was replaced with homologous sequences from across the α-integrin tree, and the percent integrin activation was determined using three replicates. EDTA reduced activation to essentially 0%. (**C**) Conformational landscapes of loop swaps from (B) reveal differential coordination of the internal ligand and α_7_ helix dynamics. Bottom: Representative trajectory snapshots of the β subunit (blue) and positional deviations of the α1 helix, α_7_ helix, and internal ligand (green). Manganese ions are purple. The MIDAS-coordinating glutamate and positional substitution are shown as sticks. Structures drawn from 25 clusters of the respective conformational landscape. WT, wild type. (**D**) Left: A schematic visualization of the flow cytometry approach used to measure ligand binding. An anti-His antibody was used as a readout for intercellular adhesion molecule–1 (ICAM-1) binding. Points represent mean, and bars are SD over three replicates. (**E**) Conformational landscapes of αEβ_7_ mutants. Representative snapshots are depicted below similar to (C). (**F**) B factor comparisons of the ligand-bound and apo αEβ_7_ structures. (**G**) The molecular structure of α_4_β_7_ shows that the internal ligand homologous motif cannot coordinate the βMIDAS, and thus, its function in the I domain background was exapted. (**H**) A model for the αI domain’s evolutionary origin. ECM, extracellular matrix.

Our finding of collagen-derived acquisition of the I domain still cannot explain the presence of the highly conserved I/LEGT motif, which contains the βMIDAS-coordinating glutamate required for internal ligand-based activation. We found no conserved matches to this motif either within collagen or within any other vWFA proteins in ProtKB metazoan genomes (Fig. 5A). Moreover, all integrin I domains lack the C-terminal cysteine, which is largely conserved in the collagen vWFA domains (Fig. 5A).

We thus hypothesized that the I/LEGT motif may have already been present in an ancestral α-integrin gene but did not incur stringent selective constraints until after it acquired the I domain. To test this possibility, we performed sequence alignments and built phylogenetic trees of the α-integrins within the deuterostome clade containing the I domain α-integrins and the most closely related I domain–lacking α_4_/α_9_ integrins (fig. S11A). We looked specifically within the region between the second and third domains of the characteristic α-integrin β propeller where the I domain was first inserted. In all clades analyzed, we found a conserved acidic residue corresponding to the essential ion-coordinating glutamate within the internal ligand, suggesting that this acidic residue was ancestrally present in the lineage of α-integrins that first acquired an I domain. Moreover, we found a VDGT motif, highly reminiscent of the I/LEGT motif, in a small cephalochordate-specific clade, which is the closest-branching I domain–lacking outgroup to the I domain α-integrins (fig. S11B).

Using a flow cytometry assay and conformation-specific antibodies, we tested whether these motifs from the non–I domain–containing α-integrins are sufficient to activate the extant I domain integrin αLβ_2_ (fig. S11C). We found that each of these motifs, or ancestral reconstruction of the entire internal ligand loop, could recapitulate ~35 to 50% of integrin activation. Furthermore, we demonstrated that this activity depended on ion coordination; mutating the acidic D/E residues to alanine in the wild-type I/LEGT or VDGT reduced activation to 20% (Fig. 5B). Moreover, adding EDTA to chelate the cation reduced activation in all backgrounds to essentially 0%. These data suggested that this cephalochordate motif was sufficient to activate integrin structurally. Conformational landscapes from molecular simulations revealed that these motif swaps are consistent with the exceptional stabilization conferred by the glutamate within the internal ligand, providing a molecular rationale for our cellular assay (Fig. 5C). VDGT is also sufficient to confer ligand-binding capability to 60% of the levels of wild type, providing a functional and structural rescue (Fig. 5D and fig. S12). MD simulations suggested that this partial rescue is consistent with partial ion coordination occupancy upon loop mutation (Fig. 5C) and behaved similarly for αEβ_7_ (Fig. 5E), revealing the dynamic basis of the essential ion-coordinating glutamate in regulating internal ligand binding and β subunit dynamics. This is further supported by B factor analyses from our αEβ_7_ structures, showing that ligand binding confers unique stability in this region, particularly E372 (Fig. 5F).

This I/LEGT-like motif is not preserved in all α-integrins. Our structure of α_4_β_7_ revealed that this loop region does not participate in ion coordination or integrin activation in I domain–lacking integrins (Fig. 5G) but instead serves to support ligand binding. Distinct non-VDGT α_4_ residues are essential for binding to vascular cell adhesion molecule–1, another α_4_β_7_ ligand (*60*). Homology modeling of the cephalochordate-specific α-integrin suggested a similar behavior in the direct outgroup (fig. S11D). Thus, the allosteric ion-coordinating internal ligand was a serendipitous molecular exaptation, which could only have occurred in some lineages of α-integrins. This exaptation enabled them to maintain foundational conformation-based signaling while acquiring new ligand-binding capabilities upon I domain insertion. Following this initial exaptation, selection continued to fine-tune α-integrins to reach their maximum activation threshold. This fine-tuning included the acquisition of the internal ligand “pocket”—a conserved structural series of buried residues capable of finely adjusting integrin kinetics (*61*)—that is highly diverged in the outgroup cephalochordate integrin (fig. S11B).

## DISCUSSION

Our analyses retrace molecular history to present a structural framework for how a large, 500-million–year–old evolutionary insertion preserved necessary protein functions while serving as a launch pad for adaptation. Despite completely occluding the integrin’s ancestral ligand-binding site, the I domain retains the same functional output of an ancestral integrin ligand at multiple levels. In this way, the I domain served two capacities right from its inception: the ability to bind to an ion-coordinating ligand and the coordination of downstream integrin conformational changes. Given the collagen origin of the I domain itself, it is likely that the I domain’s co-option into the integrin family catalyzed the ability of integrins to associate with the extracellular matrix directly (Fig. 5H). This ancestral feature (*56*, *62*) has expanded and specialized in extant integrins (*55*).

Most eukaryotic proteins contain multiple distinct structural domains (*63*), including those that contain other vWFA domains. We anticipate that the form of evolutionary molecular co-option we have uncovered has provided novelty across a wide array of molecular processes. Our analyses describe the unique and shared structural features of I domain–mediated integrin activation that were critical for its evolutionary and immunological success.

## MATERIALS AND METHODS

### DNA constructs

The regions encoding the wild-type αE ectodomain (M1-H1123; used for integrin:E-cadherin complex) followed by a C-terminal linker, HRV 3C cut site (LEVLFQGP), acidic coil motif (AQCEKELQALEKENAQLEWELQALEKELAQ), and Strep-Tag II (WSHPQFEK*) inserted into a pcDNA3.1-Hygro(−)–like backbone were synthesized commercially (GenScript). The R177G/R178G, “RRtoGG,” mutant (clasped construct used for integrin:Fab complex) was generated via site-directed mutagenesis (New England Biolabs). The region encoding the β_7_ ectodomain (M1-H723) followed by a C-terminal linker (GTGG), HRV 3C cut site (LEVLFQGP), basic coil motif (AQCKKKLQALKKKNAQLKWKLQALKKKLAQ), and 6xHis tag inserted into a pcDNA3.1-Hygro(−)–like backbone was synthesized commercially (GenScript). The region encoding the α_4_ ectodomain (M1-Q970) was amplified from Addgene plasmid #81178 and inserted into a pcDNA3.1-Hygro(−)–like backbone using standard molecular cloning techniques. The region encoding the MAdCAM-1 ectodomain (M1-Q317) was synthesized commercially as a gBlocks [Integrated DNA Technologies (IDT)] and inserted into a pcDNA3.1-Hygro(−) with the same C-terminal linker, 3C cut site, basic coil motif, and 6xHis tag as β_7_ using standard cloning techniques. The region encoding the intercellular adhesion molecule–1 (ICAM-1) ectodomain (M1-E480) was inserted into a pcDNA3. 1-Hygro(−) with the same C-terminal linker, 3C cut site, and 6xHis as for β_7_ using standard cloning techniques. For Fc-tagged E-cadherin, the region encoding the E-cadherin ectodomain (D155 to A698) with a CD33 signal sequence (GMPLLLLLPLLWAGALA) (gift from B. Gumbiner) was inserted into a pcDNA3. 1-Hygro(−)–like backbone with a C-terminal HRV 3C cut site and human Fc tag amplified from Addgene plasmid 145164 using standard cloning techniques. The bacterial expression plasmid encoding an N-terminal 6xHis-SUMO–tagged EC12 protein (E-cadherin EC domains 1 and 2; D155 to N371) was a gift from B. Gumbiner. The bacterial expression plasmids for the α_2_ (D174 to E365) and Ancestral I (AncI) domains were synthesized as gBlocks (IDT) and inserted into a pET24a vector using standard cloning techniques. The full-length wild-type αL and β_2_ constructs were made in ectodomain pcDNA3.1-Hygro(−) backbones by inserting gBlocks of the transmembrane regions by standard cloning techniques. Loop swaps of the αL IEGT internal ligand region were synthesized as gBlocks and inserted via standard cloning techniques.

### Protein expression and purification

Both integrins αEβ_7_ and α_4_β_7_, as well as MAdCAM-1 and ICAM-1, were expressed in mammalian ExpiCHO cells (Thermo Fisher Scientific). The basic expression and purification protocol is similar for each protein. Protein expression was performed according to the “Max Titer” manufacturer’s recommendations. For each integrin, a ratio of 3:2 α:β DNA was transfected for a total of 1 μg of DNA per milliliter of cell culture. For MAdCAM-1 and ICAM-1, a total of 1 μg of DNA per milliliter of cell culture was used for transfection. Cells were grown to a concentration of 7 to 10 × 10^6^ cells/ml at 37°C, 8% CO_2_, and 90% humidity. The day of transfection, cells were split to 6 × 10^6^ cells/ml and allowed to recover for 2 to 4 hours. Cells were then transfected according to the manufacturer’s recommendations using ExpiFectamine CHO and grown overnight at 37°C, 8% CO_2_, and 90% humidity. The next day, cells were given enhancer and feed according to the manufacturer’s recommendations and shifted to 32°C, 5% CO_2_, and 90% humidity to support protein expression. Five days posttransfection, cells were given a second dose of feed according to the manufacturer’s recommendations. Eight to 10 days posttransfection, cells were harvested via centrifugation for 15 min at 4°C and ×1000*g*. The cell supernatant containing secreted protein was further clarified for 20 min at 4°C and ×30,000*g* and diluted 2:1 into HisTrap Binding Buffer [20 mM NaPO_4_, 500 mM NaCl, and 20 mM imidazole (pH 7.4)]. The clarified supernatant was flowed over a 5-ml HisTrap FF Crude column (Cytiva) equilibrated in HisTrap Binding Buffer using an ÄKTA pure 25 L1 FPLC system. Bound protein was eluted in HisTrap Elution Buffer [20 mM NaPO_4_, 500 mM NaCl, and 500 mM imidazole (pH 7.4)]. Fractions containing protein according to absorbance at 280 nm (*A*_280_) were pooled, concentrated to 500 μl, and buffer exchanged into integrin storage buffer [20 mM tris-HCl (pH 7.4), 150 mM NaCl, 1 mM MgCl_2_, and 1 mM CaCl_2_] using an Amicon Ultra-15 concentrator (Millipore) with a 10-kDa cutoff. For clasped integrin proteins and ICAM-1, the concentrate was then purified via gel filtration chromatography using a Superdex 200 Increase 10/300 SEC column (Cytiva) equilibrated with integrin storage buffer. Peak fractions containing integrin according to SDS–polyacrylamide gel electrophoresis were pooled, concentrated to 2 mg/ml, snap frozen in 10% (v/v) glycerol, and stored at −80°C. For MAdCAM-1 and unclasped integrins, 1:10 (w/w) of 3C protease was added to the protein concentrate and incubated overnight with end-over-end rotation at 4°C. The next day, the cleaved protein was further purified via gel filtration chromatography, concentrated, and stored as was done with the clasped protein.

Human Fc-tagged E-cadherin ectodomain was expressed and purified similarly to integrin and MAdCAM-1 constructs. Ectodomains were expressed in ExpiCHO cells according to the “Max Titer” manufacturer’s recommendations using a total of 1 μg of DNA per milliliter of cell culture. Cells were fed and harvested, and supernatants were clarified as with the integrin constructs. Supernatant was diluted 2:1 in HiTrap binding buffer [50 mM tris-HCl (pH 7.4), 150 mM NaCl, and 3 mM CaCl_2_] and flowed over a 1-ml HiTrap Protein G HP column (Cytiva) equilibrated in HiTrap binding buffer. The column was washed with 10 volumes of HiTrap binding buffer, and protein was eluted with 100 mM glycine-HCl (pH 2.7) into 1 M tris-HCl (pH 9.0) to neutralize. Eluate was pooled and concentrated to 500 μl and was then further purified via size exclusion chromatography on a Superdex 200 Increase 10/300 SEC column (Cytiva) equilibrated in 50 mM tris-HCl (pH 7.4), 150 mM NaCl, and 3 mM CaCl_2_. Peak fractions were pooled, concentrated to 2 mg/ml, snap frozen with 10% (v/v) glycerol, and stored at −80°C.

EC12 was expressed and purified as previously described (*64*) with slight modifications. Briefly, BL21 DE3 cells containing the expression plasmid were grown to an optical density at 600 nm (OD_600_) of 0.6, induced with 0.1 mM isopropyl-β-d-thiogalactopyranoside (IPTG), and incubated with shaking overnight at 18°C. The following day, cells were harvested at ×4000*g* for 30 min at 4°C and resuspended in 10 ml/liter of cell culture in EC12 binding buffer [500 mM NaCl, 20 mM tris-HCl (pH 7.4), 3 mM CaCl_2_, and 20 mM imidazole] with EDTA-free cOmplete protease inhibitor cocktail (Roche). The cells were lysed via sonication on ice for 3 min, and the lysate was clarified for 30 min at 4°C and ×20,000*g*. The lysate was flowed over a 1-ml HisTrap FF Crude column (Cytiva) equilibrated in EC12 Binding Buffer and washed with 10 column volumes (CV) of EC12 Binding Buffer. Bound protein was eluted in 500 mM NaCl, 20 mM tris-HCl (pH 7.4), 3 mM CaCl_2_, and 250 mM imidazole. Peak fractions were pooled and concentrated using an Amicon Ultra-15 concentrator (Millipore) with a 10-kDa cutoff. The concentrate was dialyzed overnight at 4°C into 4 liters of 50 mM tris-HCl (pH 7.4), 150 mM NaCl, and 3 mM CaCl_2_ with 250 U of 6xHis-tagged SUMO protease (Thermo Fisher Scientific) for scar-free SUMO tag cleavage. The following day, the dialysate was run over a 1-ml HisTrap FF Crude column to remove the protease and unprocessed EC12. The dialysate was then further purified via gel filtration chromatography on a Superdex 75 Increase 10/300 GL (Cytiva). Peak fractions containing EC12 were pooled, concentrated to 2 mg/ml, and snap frozen in 10% (v/v) glycerol. We attempted to express C-terminally His-tagged EC12 in mammalian cell culture but proteins consistently precipitated following HisTrap elution.

Recombinant 6xHis-tagged integrin I domains (α_2_ and AncI) were expressed and purified from BL21 DE3 LOBSTR cells. Cells containing the expression plasmid were grown to an OD_600_ of 0.6, induced with 0.1 mM IPTG, and incubated with shaking overnight at 18°C. The following day cells were harvested at ×4000*g* for 30 min at 4°C and resuspended in 10 ml/liter of cell culture in 20 mM NaPO_4_, 500 mM NaCl, 20 mM imidazole (pH 7.4), 1% Triton X-100, EDTA-free cOmplete protease inhibitor cocktail (Roche), and 0.5 mM phenylmethylsulfonyl fluoride added immediately before resuspension. Following an hour of 4°C incubation, the lysate was flowed over a 1-ml HisTrap Excel column (Cytiva) equilibrated in 20 mM NaPO_4_, 500 mM NaCl, and 20 mM imidazole (pH 7.4). I domains were eluted in 20 mM NaPO_4_, 500 mM NaCl, and 500 mM imidazole (pH 7.4) in 1-ml fractions. Peak fractions were pooled, concentrated to 500 μl using an Amicon Ultra-15 concentrator (Millipore) with a 10-kDa cutoff, and further purified or analyzed via gel filtration chromatography on a Superdex 200 Increase 10/300 GL (Cytiva) equilibrated in integrin storage buffer. I domain–containing fractions were concentrated to 10 mg/ml in integrin storage buffer, snap-frozen in 10% (v/v) glycerol, and stored at −80°C.

### nsEM sample preparation

Integrin αEβ_7_ was diluted to a final concentration of between 2 and 20 μg/ml in buffer containing 20 mM tris-HCl (pH 7.4), 150 mM NaCl, and ion concentrations indicated in fig. S4. Integrin (3 μl) was applied to a glow-discharged 400 mesh copper gilder grid (Ted Pella) that had been covered with a thin layer of continuous amorphous carbon. AncI was diluted to 10 μg/ml in integrin storage buffer and applied to grids in a similar way to αEβ_7_. The grids were stained with a solution containing 2% (w/v) uranyl formate as previously described (*65*).

### nsEM data acquisition and processing

Data were acquired using a Thermo Fisher Scientific Talos L120C transmission electron microscope operating at 120 kV and recorded on a 4096 pixel by 4096 pixel Thermo Fisher Scientific Ceta camera at a nominal magnification of ×92,000 with a pixel size of 0.158 nm. Leginon (*66*) was used to collect 373 (αEβ_7_; 5 mM CaCl_2_), 373 (αEβ_7_; 1 mM MgCl_2_ and 1 mM CaCl_2_), 320 (αEβ_7_; 1 mM MnCl_2_), or 311 (AncI) micrographs at a nominal range of 1.5- to 2.5-μm underfocus and a dose of ~50 e^−^/Å^2^.

Similar processing pipelines were used for all negative-stain datasets. Micrographs were processed using cryoSPARC (*67*). Cross Transfer Function (CTF) estimation was done using Patch CTF. Initially, 758,281 (αEβ_7_; 5 mM CaCl_2_), 736,876 (αEβ_7_; 1 mM MgCl_2_ and 1 mM CaCl_2_), 629,410 (αEβ_7_; 1 mM MnCl_2_), or 621,999 (AncI) particles were picked in a reference-free manner using blob picker in cryoSPARC. The particle picks were subjected to one round of particle curation and one round of 2D classification, followed by 2D class selection for a final particle count of 17,971 (5 mM CaCl_2_), 9092 (1 mM MgCl_2_ and 1 mM CaCl_2_), 18,552 (1 mM MnCl_2_), or 56,638 (AncI) contributing to 2D classes. The 10 clearest integrin classes for each buffer condition were chosen on the basis of the visibility of the I domain and headpiece region.

### cryo-EM sample preparation

The apo α_4_β_7_ protein was diluted 1:10 in nonactivating buffer [20 mM tris-HCl (pH 7.4), 150 mM NaCl, and 5 mM CaCl_2_] to a final concentration of 0.2 mg/ml. The RRtoGG αEβ_7_ ectodomain was diluted 1:10 in nonactivating buffer and incubated with digested LF61 Fab (Invitrogen) at a 1:4 molar ratio for 45 min at room temperature with end-over-end mixing. The integrin:Fab complex was subjected to size exclusion chromatography in nonactivating buffer, and complex peaks were concentrated to 0.25 mg/ml. The wild-type αEβ_7_ ectodomain was diluted 1:10 in activating buffer [20 mM tris-HCl (pH 7.4), 150 mM NaCl, and 1 mM MnCl_2_] (*38*, *51*, *68*, *69*) and incubated with EC12 at a 1:4 molar ratio for 45 min at room temperature with end-over-end mixing. The integrin:ligand complex was subjected to size exclusion chromatography in activating buffer, and complex peaks were concentrated to 1 mg/ml. Immediately before freezing, stock CHAPS detergent was added to the integrin:ligand complex to a final concentration of 0.05% (v/v). The α_4_β_7_:MAdCAM-1 complex was formed and prepared in the same way as αEβ_7_:EC12. Following dilution and complex formation steps, all samples were frozen under similar conditions. Protein (3 μl) was applied to QUANTIFOIL grids (Electron Microscopy Sciences) that were glow discharged for 30 s at 15 mA. The grids were blotted with a Vitrobot Mark IV (Thermo Fisher Scientific) using a blot time of 3 to 7 s and blot force of 4 to 5 at 100% humidity and 4°C. Grids were plunge frozen in liquid ethane cooled by liquid nitrogen and stored in liquid nitrogen.

### Cryo-EM data acquisition and processing

The details of datasets and processing pipelines are outlined in table S1, figs. S1 (α_4_β_7_:MAdCAM-1), S3 (apo α_4_β_7_), S5 and S6 (αEβ_7_:LF61 Fab), and S7 (αEβ_7_:EC12). All datasets were collected on a Glacios cryo–transmission electron microscope (Thermo Fisher Scientific) operating at 200 kV and recorded with a Gatan K3 Direct Detection Camera. Automated data collection was carried out using SerialEM (*70*). One hundred-frame movies were recorded in superresolution counting mode at a nominal magnification of ×36,000 corresponding to a calibrated superresolution pixel size of 0.561 Å per pixel. Each dataset was collected with a nominal defocus range of 1.2 to 1.8 μm underfocus and a dose of ~50 e^−^/Å^2^.

Dose-fractionated superresolution movies were motion corrected and binned 2 × 2 by Fourier cropping using MotionCor2 (*71*) within the RELION (*72*) wrapper. From there, motion-corrected stacks were further processed in cryoSPARC according to each processing pipeline. Briefly, particles were picked using the blob picker, subjected to multiple rounds of 2D and/or 3D classification using ab initio and heterogeneous refinement using initial models generated from the data, and further refined with nonuniform and local refinement. Masks for local refinements were generated using UCSF ChimeraX (*73*) and cryoSPARC. Map sharpening was performed using the COSMIC2 (*74*) server wrapper of DeepEMhancer (*75*) with the high resolution setting. Local resolution estimation was performed in cryoSPARC. 3D Fourier shell correlation (*76*) plots were generated from https://3dfsc.salk.edu/.

CryoSPARC was used for all motion and variability analyses. We used the cluster setting in 3D variability analysis (*39*), followed by homogeneous and heterogeneous refinement to sort the αEβ_7_:LF61 Fab particles into open or closed I domain states. Maps showing that the conformational range of the internal-liganded β_7_ leg in the Fab-bound structure (Fig. 2G) were generated using 3D classification in principal components analysis mode. We generated the volume series showing the continuous motion of the β_7_ in ligand-bound structures using 3DFlex (*77*). All map visualizations (images and movies) were generated and recorded using UCSF ChimeraX.

### Model building

For the Fab-bound initial αEβ_7_ model, we used an AlphaFold2 (*54*) model of αE and PDB 3V4P (*20*), chain B for β_7_. We predicted the LF61 Fab residues using ModelAngelo (*78*) and fit them into mouse IgG1 constant residue sequences. The initial models were manually fit into the density using UCSF ChimeraX, followed by dock-in-map in Phenix (*79*). For the initial αEβ_7_:EC12 model, we used the AlphaFold2 αE model, a homology model of β_7_ built in SWISS-MODEL (*80*) using PDB 7NWL (*23*), chain B as the reference, and PDB 4ZT1 (*81*), chain A for EC12. We generated the initial α_4_β_7_:MAdCAM-1 model using AlphaFold-Multimer (*82*).

Models were manually adjusted in an iterative way between Coot (*83*) and ISOLDE (*84*) within UCSF ChimeraX. The β_7_ hybrid domain was flexibly fit into the ligand-bound models using ISOLDE. The αEβ_7_:LF61 Fab model was refined using the closed I domain map. Glycans were built manually in Coot using the “carbohydrate” module. Models were built using a combination of the nonuniform and locally refined, unsharpened and sharpened maps. Rosetta (*85*) was used to depict secondary structure. All maps used for model building have been deposited.

Electrostatic molecular interactions were determined using the PISA server (*86*). RMSD calculations for the βI domain were done in ChimeraX using the “matchmaker” function with β_7_ residues V133 to S369.

B factor analyses were done as in Jin *et al.* (*87*). Briefly, B factors were calculated from Phenix Real-space Refine (*79*) and normalized to the most stable region of the αE headpiece, the β propeller. Graphics were generated by coloring models by B factors in UCSF ChimeraX.

### System setup for MD simulations

To understand the ancestral conformational dynamics of the integrin I domain, we simulated a representative sample of integrin I domains (αE, αL, α_2_, and *Petromyzon marinus* XP_032834731), sister clade collagen-derived vWFA domains (co6a6 800 to 988, co6a5 433 to 618, coea1 1024 to 1210, and VWA1 29 to 211), RPN10 to be used as a distant outgroup, and the ancestral reconstruction AncI (described in the following sections). The initial coordinates of the studied I domains were obtained from the cryo-EM model (αE: this study), prior crystal structures [αL: 3F74 (*88*), α_2_: 5HJ2 (*89*), and RPN10: 5LN1 (*90*)], and AlphaFold2 (XP_032834731)– or AlphaFold3 (*91*)–predicted models (co6a6 800 to 988, co6a5 433 to 618, coea1 1024 to 1210, and VWA1 29 to 211). AlphaFold3-predicted models were generated using the online web server (alphafoldserver.com), and AlphaFold2-predicted models were generated in ColabFold (*92*). The proteins were solvated in a periodic truncated octahedron with Optimal Point Charge (OPC) water molecules and 150 mM NaCl. One magnesium, manganese, or calcium ion was placed in the αMIDAS site. For RPN10, no additional ion was modeled.

To investigate the effect of amino acid substitution on the internal ligand, we modeled truncated integrin systems of αLβ_2_ bound with ICAM-1 and αEβ_7_ bound to E-cadherin. In all, the truncated models contain the respective α subunit β propeller, α subunit I domain, and β-subunit I domain. The initial model for αLβ_2_ was generated by combining SWISS-MODEL homology models of the open β_2_ subunit using β_7_ as a template (this study), the open αL subunit using PDB 4NEH (*10*) as a template, and the ICAM-1–bound αL I domain [PDB 1MQ8 (*49*)]. The cryo-EM structure αEβ_7_ bound to E-cadherin was used as the initial model. Both systems were solvated in a periodic truncated octahedron with OPC water molecules and 150 mM NaCl. Four manganese ions were added to the αMIDAS, MIDAS, ADMIDAS, and SyMBS coordinating sites. Three calcium ions were added to the calcium-binding sites of the α subunit.

### MD protocols

All simulations were performed using OpenMM 7.7.0 (I-domain systems) (*93*) or OpenMM 8.2.0 (*94*) (integrin headpiece systems) using AMBER ff19SB force field (*95*), OPC water model, and 12-6-4 Li Merz ion parameters (*96*). Before production, systems were energy minimized for 20,000 steps and equilibrated at 298 K (I-domain systems) or 310 K (integrin headpiece systems) with backbone atoms harmonically restrained by a force constant (5 kcal/mol × Å^2^) for 10 ns. Langevin dynamics was performed using a Langevin integrator with an integration timestep of 4 fs and a collision rate of √2 ps^−1^ and hydrogen mass repartitioning (*97*). Pressure was maintained using a Monte Carlo barostat with an update frequency of 100 steps. Nonbonded interactions were calculated with a distance cutoff of 10 Å. Trajectory snapshots were saved every 100 ps during production simulations. Trajectory lengths and number of replicates of each simulated system are listed in table S2. Simulations were performed on either the Fred Hutch Cancer Center clusters on NVIDIA GTX 2080 or in-house workstations on NVIDIA GTX 3090.

Trajectories were processed using in-house scripts by CPPTRAJ 6.18.1 (*98*) and MDTraj 1.10.2 (*99*) packages. For Fig. 4C and fig. S10, RMSD and root mean square fluctuation (RMSF) measurements were performed on Cα atoms using the initial structure as the reference and considered the last 2.0 μs of each simulation trajectory. For Fig. 4D, UMAP dimensionality reduction was performed using the UMAP 0.5.6 Python package (*100*). Backbone torsion angles (phi, psi, and omega) were used as features for UMAP with the following parameters: n_neighbors = 200 and max_dist = 0.99. For Fig. 5 (C and E), the last 0.8 μs of production simulations were considered. To calculate positional displacement (no-fit RMSD) of the internal ligand and helix α_7_ of the β subunit, structures were first superpositioned by Cα of the β subunit. For the internal ligand, the Cα atoms for two residues before and after the MIDAS-coordinating glutamate residue (or equivalent position; five residues total) were considered for RMSD analysis. For helix α_7_ of the β subunit, β_2_ residues 348 to 363 or β_7_ residues 375 to 389 Cα atoms were used. Twenty-five clusters from each conformational landscape were generated using a KMeans algorithm implemented in scikit-learn 1.5.0 (fig. S12).

### Biolayer interferometry

Assays were performed on an Octet RED (ForteBio) instrument at 25°C with shaking at 1000 rpm. Protein A biosensors were hydrated in activating buffer with the addition of 0.1% bovine serum albumin and 0.02% Tween 20 (BLI buffer) for 15 min. Fc-tagged E-cadherin ectodomain was loaded at 20 μg/ml in BLI buffer until a threshold of 1.2 nm was reached. A baseline equilibration step was performed in BLI buffer for 2 min. Association of αEβ_7_ in BLI buffer at various concentrations in a two-fold dilution series from 500 to 16.125 nM was carried out for 600 s before dissociation. The data were baseline subtracted before fitting was performed using a 1:1 binding model and the ForteBio data analysis software. Mean *K*_d_ values were determined with a global fit applied to all data from three independent replicates. A representative sensorgram of the three replicates is displayed in the relevant figure.

### Sequence curation and HMM searching

Protein sequences for all human integrin subunits were curated in the National Center for Biotechnology Information (NCBI) database wrapper within Geneious Prime. Representative I domain sequences from tunicates were curated using BLAST with a urochordate taxon restriction and human αL as a search query. HMM searching of UniProtKB was performed on the online HMMER server from the European Bioinformatics Institute (*101*) with the full integrin I domain alignment as an initial query. Because no sequences containing the internal ligand I/LEGT motif were found, we restricted our search to exclude this region of the I domain alignment and included only results from representative species of human, coelacanth, echinoderms, and *Branchiostoma*. Sequences with an individual *E* value of less than 1 × 10^–30^ were considered hits and used for subsequent alignment and phylogenetic analyses.

### Sequence alignments

All protein sequence alignments were generated using the default Multiple Sequence Comparison by Log-Expectation (MUSCLE) algorithm (*102*) and visualized using Geneious Prime. Alignments were further curated manually to remove large gaps and sequences with low quality. Sequence alignments are available as supplemental text files in FASTA or PHYLIP format with the following names: alignment of all human α subunits (data S1) except αE, which was excluded because of an extra “X” domain preceding the I domain that is unique to αE, alignment of integrin I domains across humans and tunicates used for HMM search and ancestor reconstruction (data S3), alignment of all hits from HMM search (data S4), and alignment of expanded integrin clade encompassing I domain integrins and near neighbors (data S5). Sequence names are provided as protein names, NCBI accession numbers, and UniProt, Ensembl identifiers, or ANISEED transcript IDs.

### Flow cytometry

Human embryonic kidney (HEK) 293T cells (HEK293T cells) were passaged in 10-cm tissue culture–treated plates every 2 to 3 days in Dulbecco’s modified Eagle’s medium + l-glutamine supplemented with 10% fetal bovine serum and 1% penicillin-streptomycin. For integrin activation and ligand-binding experiments, HEK293T cells were transfected in six-well tissue culture–treated plates at 70% confluence using Lipofectamine 2000 according to the manufacturer’s instructions. Each well was transfected with 2.5 μg of a 1.5:1 ratio mixture of DNA encoding the αL variants and β_2_ wild-type genes, respectively. Cells were washed 24 hours after transfection, and fresh medium was applied. Cells were trypsinized and washed 36 hours after transfection and harvested for subsequent experiments.

For integrin activation experiments, each well of cells was centrifuged for 5 min at ×500*g*, washed with warmed 20 mM tris-HCl (pH 7.4), 150 mM NaCl, centrifuged again, and resuspended in 500 μl of either 20 mM tris-HCl (pH 7.4), 150 mM NaCl, and 5 mM EDTA or 20 mM tris-HCl (pH 7.4), 150 mM NaCl, and 1 mM MgCl_2_ + 1 mM MnCl_2_. Following a 30-min rocking incubation at 37°C, 1 μl of each fluorescein isothiocyanate (FITC) TS2/4 and APC m24 antibody (BioLegend) was added to each cell suspension. Cells were incubated for 1 hour, washed with 1 ml and resuspended in 100 μl of the appropriate buffer, and placed on ice for flow analysis. A total of >10,000 cells for each condition were analyzed on either a BD FACSCanto II or FACSymphony A5. The presence of FITC and APC was measured on the basis of an unstained wild-type integrin control from the same six-well plate. Each condition was repeated three times.

For ligand-binding assays, 2 × 10^6^ cells from each well were centrifuged for 5 min at ×500*g*, washed with warmed 20 mM tris-HCl (pH 7.4), 150 mM NaCl, centrifuged again, and resuspended in 500 μl of 20 mM tris-HCl (pH 7.4), 150 mM NaCl, and 1 mM MgCl_2_ + 1 mM MnCl_2_. Following a 30-min rocking incubation at 37°C, purified His-tagged ICAM-1 ectodomain was added to each cell suspension to a final concentration of 20 μg/ml. Cells were incubated for another hour at 37°C, washed with ice-cold buffer, and resuspended in 100 μl buffer with 1 μl of each FITC TS2/4 and iFluor 647 anti-His tag antibody. Following a 1-hour incubation at 4°C, cells were washed with ice-cold buffer and resuspended on ice in 100 μl buffer. A total of >10,000 cells for each condition were analyzed on a BD FACSCanto II. The presence of FITC and iFluor647 was measured on the basis of an unstained wild-type integrin control from the same six-well plate. The percentage of iFluor 647–positive cells was normalized to the wild-type control. Each condition was repeated three times.

### Phylogenetic analysis

All phylogenetic trees were generated using a maximum likelihood framework within IQ-TREE (*103*). Substitution models for each alignment were estimated with IQ-TREE. Both 1000 ultrafast bootstrap and SH-aLRT replicates are displayed as branch support statistics, also generated in IQ-TREE. Trees were visualized in FigTree (*104*).

### Ancestral sequence reconstruction

To reconstruct the ancestral I domain sequence, we curated and manually edited an amino acid alignment of I domains from integrins in both vertebrate and urochordate lineages with the closely related cephalochordate collagen vWFA domain as the outgroup. Maximum likelihood–based ancestral reconstruction was carried out using the codeml package in PAML 4.8 (*53*), and the marginal reconstruction at each site was used as the AncI sequence.

## References

[1] M. Bashton, C. Chothia, The generation of new protein functions by the combination of domains. Structure 15, 85–99 (2007). 17223535 10.1016/j.str.2006.11.009

[2] Y. Takada, X. Ye, S. Simon, The integrins. Genome Biol. 8, 215 (2007). 17543136 10.1186/gb-2007-8-5-215 PMC1929136

[3] R. O. Hynes, Integrins: Bidirectional, allosteric signaling machines. Cell 110, 673–687 (2002). 12297042 10.1016/s0092-8674(02)00971-6

[4] M. S. Johnson, N. Lu, K. Denessiouk, J. Heino, D. Gullberg, Integrins during evolution: Evolutionary trees and model organisms. Biochim. Biophys. Acta 1788, 779–789 (2009). 19161977 10.1016/j.bbamem.2008.12.013

[5] A. Kern, R. Briesewitz, I. Bank, E. E. Marcantonio, The role of the I domain in ligand binding of the human integrin alpha 1 beta 1. J. Biol. Chem. 269, 22811–22816 (1994). 7521332

[6] C. Huang, T. A. Springer, Folding of the β-propeller domain of the integrin α_L_ subunit is independent of the I domain and dependent on the β_2_ subunit. Proc. Natl. Acad. Sci. U.S.A. 94, 3162–3167 (1997). 9096363 10.1073/pnas.94.7.3162 PMC20339

[7] M. S. Johnson, B. S. Chouhan, Evolution of integrin I domains. Adv. Exp. Med. Biol. 819, 1–19 (2014). 25023164 10.1007/978-94-017-9153-3_1

[8] E. Ruoslahti, RGD and other recognition sequences for integrins. Annu. Rev. Cell Dev. Biol. 12, 697–715 (1996). 8970741 10.1146/annurev.cellbio.12.1.697

[9] B. LaFoya, J. A. Munroe, A. Miyamoto, M. A. Detweiler, J. J. Crow, T. Gazdik, A. R. Albig, Beyond the matrix: The many non-ECM ligands for integrins. Int. J. Mol. Sci. 19, 449 (2018). 29393909 10.3390/ijms19020449 PMC5855671

[10] M. Sen, K. Yuki, T. A. Springer, An internal ligand-bound, metastable state of a leukocyte integrin, α_X_β_2_. J. Cell Biol. 203, 629–642 (2013). 24385486 10.1083/jcb.201308083 PMC3840939

[11] G. Gorfu, J. Rivera-Nieves, K. Ley, Role of β_7_ integrins in intestinal lymphocyte homing and retention. Curr. Mol. Med. 9, 836–850 (2009). 19860663 10.2174/156652409789105525 PMC2770881

[12] S. K. Shaw, M. B. Brenner, The β_7_ integrins in mucosal homing and retention. Semin. Immunol. 7, 335–342 (1995). 8580465 10.1016/1044-5323(95)90014-4

[13] P. J. Rutgeerts, R. N. Fedorak, D. W. Hommes, A. Sturm, D. C. Baumgart, B. Bressler, S. Schreiber, J. C. Mansfield, M. Williams, M. Tang, J. Visich, X. Wei, M. Keir, D. Luca, D. Danilenko, J. Egen, S. O’Byrne, A randomised phase I study of etrolizumab (rhuMAb β7) in moderate to severe ulcerative colitis. Gut 62, 1122–1130 (2013). 22717454 10.1136/gutjnl-2011-301769 PMC3711369

[14] B. Dai, J. A. Hackney, R. Ichikawa, A. Nguyen, J. Elstrott, L. D. Orozco, K.-H. Sun, Z. Modrusan, A. Gogineni, A. Scherl, J. Gubatan, A. Habtezion, M. Deswal, M. Somsouk, W. A. Faubion, A. Chai, Z. Sharafali, A. Hassanali, Y. S. Oh, S. Tole, J. McBride, M. E. Keir, T. Yi, Dual targeting of lymphocyte homing and retention through α4β7 and αEβ7 inhibition in inflammatory bowel disease. Cell Rep. Med. 2, 100381 (2021). 34467254 10.1016/j.xcrm.2021.100381 PMC8385326

[15] P. Eder, M. Kłopocka, H. Cichoż-Lach, R. Talar-Wojnarowska, M. Kopertowska-Majchrzak, A. Michalak, R. Filip, K. Waszak, K. Stawczyk-Eder, M. Janiak, K. Skrobot, A. Liebert, H. Zatorski, A. Solarska-Półchłopek, M. Krogulecki, A. Pękała, E. Poniewierka, I. Smoła, A. Kaczka, K. Wojciechowski, S. Drygała, E. Zagórowicz, Real-world outcomes of 54-week vedolizumab therapy and response durability after treatment discontinuation in ulcerative colitis: Results from a multicenter prospective POLONEZ study. Therap. Adv. Gastroenterol. 16, 17562848231151295 (2023). 10.1177/17562848231151295 PMC9932778 36818601

[16] C. Berlin, E. L. Berg, M. J. Briskin, D. P. Andrew, P. J. Kilshaw, B. Holzmann, I. L. Weissman, A. Hamann, E. C. Butcher, α4β7 integrin mediates lymphocyte binding to the mucosal vascular addressin MAdCAM-1. Cell 74, 185–195 (1993). 7687523 10.1016/0092-8674(93)90305-a

[17] N. Green, J. Rosebrook, N. Cochran, K. Tan, J. H. Wang, T. A. Springer, M. J. Briskin, Mutational analysis of MAdCAM-1/α4β7 interactions reveals significant binding determinants in both the first and second immunuglobulin domains. Cell Adhes. Commun. 7, 167–181 (1999). 10626902 10.3109/15419069909010800

[18] J. Takagi, T. A. Springer, Integrin activation and structural rearrangement. Immunol. Rev. 186, 141–163 (2002). 12234369 10.1034/j.1600-065x.2002.18613.x

[19] J. Takagi, B. M. Petre, T. Walz, T. A. Springer, Global conformational rearrangements in integrin extracellular domains in outside-in and inside-out signaling. Cell 110, 599–611 (2002). 12230977 10.1016/s0092-8674(02)00935-2

[20] Y. Yu, J. Zhu, L.-Z. Mi, T. Walz, H. Sun, J. Chen, T. A. Springer, Structural specializations of α_4_β_7_, an integrin that mediates rolling adhesion. J. Cell Biol. 196, 131–146 (2012). 22232704 10.1083/jcb.201110023 PMC3255974

[21] G. Bazzoni, L. Ma, M. L. Blue, M. E. Hemler, Divalent cations and ligands induce conformational changes that are highly divergent among β_1_ integrins. J. Biol. Chem. 273, 6670–6678 (1998). 9506964 10.1074/jbc.273.12.6670

[22] J. Takagi, K. Strokovich, T. A. Springer, T. Walz, Structure of integrin α_5_β_1_ in complex with fibronectin. EMBO J. 22, 4607–4615 (2003). 12970173 10.1093/emboj/cdg445 PMC212714

[23] S. Schumacher, D. Dedden, R. V. Nunez, K. Matoba, J. Takagi, C. Biertümpfel, N. Mizuno, Structural insights into integrin α_5_β_1_ opening by fibronectin ligand. Sci. Adv. 7, eabe9716 (2021). 33962943 10.1126/sciadv.abe9716 PMC8104898

[24] J. Dando, K. W. Wilkinson, S. Ortlepp, D. J. King, R. L. Brady, A reassessment of the MAdCAM-1 structure and its role in integrin recognition. Acta Crystallogr. D Biol. Crystallogr. 58, 233–241 (2002). 11807247 10.1107/s0907444901020522

[25] K. Tan, J. M. Casasnovas, J.-H. Liu, M. J. Briskin, T. A. Springer, J.-H. Wang, The structure of immunoglobulin superfamily domains 1 and 2 of MAdCAM-1 reveals novel features important for integrin recognition. Structure 6, 793–801 (1998). 9655832 10.1016/s0969-2126(98)00080-x

[26] B. S. Ludwig, H. Kessler, S. Kossatz, U. Reuning, RGD-binding integrins revisited: How recently discovered functions and novel synthetic ligands (re-)shape an ever-evolving field. Cancers 13, 1711 (2021). 33916607 10.3390/cancers13071711 PMC8038522

[27] J. Chen, A. Salas, T. A. Springer, Bistable regulation of integrin adhesiveness by a bipolar metal ion cluster. Nat. Struct. Biol. 10, 995–1001 (2003). 14608374 10.1038/nsb1011

[28] J. L. Viney, S. Jones, H. H. Chiu, B. Lagrimas, M. E. Renz, L. G. Presta, D. Jackson, K. J. Hillan, S. Lew, S. Fong, Mucosal addressin cell adhesion molecule-1: A structural and functional analysis demarcates the integrin binding motif. J. Immunol. 157, 2488–2497 (1996). 8805649

[29] C. Berlin, R. F. Bargatze, J. J. Campbell, U. H. von Andrian, M. C. Szabo, S. R. Hasslen, R. D. Nelson, E. L. Berg, S. L. Erlandsen, E. C. Butcher, α4 integrins mediate lymphocyte attachment and rolling under physiologic flow. Cell 80, 413–422 (1995). 7532110 10.1016/0092-8674(95)90491-3

[30] G. J. Russell, C. M. Parker, K. L. Cepek, D. A. Mandelbrot, A. Sood, E. Mizoguchi, E. C. Ebert, M. B. Brenner, A. K. Bhan, Distinct structural and functional epitopes of the α^E^β_7_ integrin. Eur. J. Immunol. 24, 2832–2841 (1994). 7525307 10.1002/eji.1830241138

[31] P. A. Szabo, M. Miron, D. L. Farber, Location, location, location: Tissue resident memory T cells in mice and humans. Sci. Immunol. 4, eaas9673 (2019). 30952804 10.1126/sciimmunol.aas9673 PMC6778482

[32] J. M. Schenkel, D. Masopust, Tissue-resident memory T cells. Immunity 41, 886–897 (2014). 25526304 10.1016/j.immuni.2014.12.007 PMC4276131

[33] S. L. Park, T. Gebhardt, L. K. Mackay, Tissue-resident memory T cells in cancer immunosurveillance. Trends Immunol. 40, 735–747 (2019). 31255505 10.1016/j.it.2019.06.002

[34] R. A. Clark, Resident memory T cells in human health and disease. Sci. Transl. Med. 7, 269rv1 (2015). 10.1126/scitranslmed.3010641 PMC4425129 25568072

[35] H. Akasaka, D. Sato, W. Shihoya, O. Nureki, Y. Kise, Cryo-EM structure of I domain-containing integrin αEβ7. Biochem. Biophys. Res. Commun. 721, 150121 (2024). 38781659 10.1016/j.bbrc.2024.150121

[36] R. K. Jensen, H. Pedersen, J. Lorentzen, N. S. Laursen, T. Vorup-Jensen, G. R. Andersen, Structural insights into the function-modulating effects of nanobody binding to the integrin receptor α_M_β_2_. J. Biol. Chem. 298, 102168 (2022). 35738398 10.1016/j.jbc.2022.102168 PMC9287160

[37] J. L. Alonso, M. Essafi, J. P. Xiong, T. Stehle, M. A. Arnaout, Does the integrin αA domain act as a ligand for its βA domain? Curr. Biol. 12, R340–R342 (2002). 12015130 10.1016/s0960-9822(02)00852-7

[38] J. O. Lee, L. A. Bankston, M. A. Arnaout, R. C. Liddington, Two conformations of the integrin A-domain (I-domain): A pathway for activation? Structure 3, 1333–1340 (1995). 8747460 10.1016/s0969-2126(01)00271-4

[39] A. Punjani, D. J. Fleet, 3D variability analysis: Resolving continuous flexibility and discrete heterogeneity from single particle cryo-EM. J. Struct. Biol. 213, 107702 (2021). 33582281 10.1016/j.jsb.2021.107702

[40] K. L. Cepek, S. K. Shaw, C. M. Parker, G. J. Russell, J. S. Morrow, D. L. Rimm, M. B. Brenner, Adhesion between epithelial cells and T lymphocytes mediated by E-cadherin and the α^E^β_7_ integrin. Nature 372, 190–193 (1994). 7969453 10.1038/372190a0

[41] P. I. Karecla, S. J. Green, S. J. Bowden, J. Coadwell, P. J. Kilshaw, Identification of a binding site for integrin αEβ_7_ in the N-terminal domain of E-cadherin. J. Biol. Chem. 271, 30909–30915 (1996). 8940076 10.1074/jbc.271.48.30909

[42] J. M. Higgins, D. A. Mandlebrot, S. K. Shaw, G. J. Russell, E. A. Murphy, Y. T. Chen, W. J. Nelson, C. M. Parker, M. B. Brenner, Direct and regulated interaction of integrin α_E_β_7_ with E-cadherin. J. Cell Biol. 140, 197–210 (1998). 9425167 10.1083/jcb.140.1.197 PMC2132596

[43] S. Troyanovsky, Cadherin dimers in cell-cell adhesion. Eur. J. Cell Biol. 84, 225–233 (2005). 15819403 10.1016/j.ejcb.2004.12.009

[44] J. M. Higgins, M. Cernadas, K. Tan, A. Irie, J. Wang, Y. Takada, M. B. Brenner, The role of α and β chains in ligand recognition by β_7_ integrins. J. Biol. Chem. 275, 25652–25664 (2000). 10837471 10.1074/jbc.M001228200

[45] J. Vendome, S. Posy, X. Jin, F. Bahna, G. Ahlsen, L. Shapiro, B. Honig, Molecular design principles underlying β-strand swapping in the adhesive dimerization of cadherins. Nat. Struct. Mol. Biol. 18, 693–700 (2011). 21572446 10.1038/nsmb.2051 PMC3113550

[46] C. P. Chen, S. Posy, A. Ben-Shaul, L. Shapiro, B. H. Honig, Specificity of cell-cell adhesion by classical cadherins: Critical role for low-affinity dimerization through beta-strand swapping. Proc. Natl. Acad. Sci. U.S.A. 102, 8531–8536 (2005). 15937105 10.1073/pnas.0503319102 PMC1150851

[47] J. Emsley, C. G. Knight, R. W. Farndale, M. J. Barnes, R. C. Liddington, Structural basis of collagen recognition by integrin α2β1. Cell 101, 47–56 (2000). 10778855 10.1016/S0092-8674(00)80622-4

[48] F. J. Fernández, J. Santos-López, R. Martínez-Barricarte, J. Querol-García, H. Martín-Merinero, S. Navas-Yuste, M. Savko, W. E. Shepard, S. Rodríguez de Córdoba, M. C. Vega, The crystal structure of iC3b-CR3 αI reveals a modular recognition of the main opsonin iC3b by the CR3 integrin receptor. Nat. Commun. 13, 1955 (2022). 35413960 10.1038/s41467-022-29580-2 PMC9005620

[49] M. Shimaoka, T. Xiao, J.-H. Liu, Y. Yang, Y. Dong, C.-D. Jun, A. McCormack, R. Zhang, A. Joachimiak, J. Takagi, J.-H. Wang, T. A. Springer, Structures of the αL I domain and its complex with ICAM-1 reveal a shape-shifting pathway for integrin regulation. Cell 112, 99–111 (2003). 12526797 10.1016/s0092-8674(02)01257-6 PMC4372089

[50] C. A. Whittaker, R. O. Hynes, Distribution and evolution of von Willebrand/integrin A domains: Widely dispersed domains with roles in cell adhesion and elsewhere. Mol. Biol. Cell 13, 3369–3387 (2002). 12388743 10.1091/mbc.E02-05-0259 PMC129952

[51] K. Zhang, J. Chen, The regulation of integrin function by divalent cations. Cell Adh. Migr. 6, 20–29 (2012). 22647937 10.4161/cam.18702 PMC3364134

[52] H. Solomon-Degefa, J. M. Gebauer, C. M. Jeffries, C. D. Freiburg, P. Meckelburg, L. E. Bird, U. Baumann, D. I. Svergun, R. J. Owens, J. M. Werner, E. Behrmann, M. Paulsson, R. Wagener, Structure of a collagen VI α3 chain VWA domain array: Adaptability and functional implications of myopathy causing mutations. J. Biol. Chem. 295, 12755–12771 (2020). 32719005 10.1074/jbc.RA120.014865 PMC7476709

[53] Z. Yang, PAML 4: Phylogenetic analysis by maximum likelihood. Mol. Biol. Evol. 24, 1586–1591 (2007). 17483113 10.1093/molbev/msm088

[54] J. Jumper, R. Evans, A. Pritzel, T. Green, M. Figurnov, O. Ronneberger, K. Tunyasuvunakool, R. Bates, A. Žídek, A. Potapenko, A. Bridgland, C. Meyer, S. A. A. Kohl, A. J. Ballard, A. Cowie, B. Romera-Paredes, S. Nikolov, R. Jain, J. Adler, T. Back, S. Petersen, D. Reiman, E. Clancy, M. Zielinski, M. Steinegger, M. Pacholska, T. Berghammer, S. Bodenstein, D. Silver, O. Vinyals, A. W. Senior, K. Kavukcuoglu, P. Kohli, D. Hassabis, Highly accurate protein structure prediction with AlphaFold. Nature 596, 583–589 (2021). 34265844 10.1038/s41586-021-03819-2 PMC8371605

[55] M. Tulla, O. T. Pentikäinen, T. Viitasalo, J. Käpylä, U. Impola, P. Nykvist, L. Nissinen, M. S. Johnson, J. Heino, Selective binding of collagen subtypes by integrin α_1_I, α_2_I, and α_10_I domains. J. Biol. Chem. 276, 48206–48212 (2001). 11572855 10.1074/jbc.M104058200

[56] M. Tulla, M. Huhtala, J. Jäälinoja, J. Käpylä, R. W. Farndale, L. Ala-Kokko, M. S. Johnson, J. Heino, Analysis of an ascidian integrin provides new insight into early evolution of collagen recognition. FEBS Lett. 581, 2434–2440 (2007). 17485091 10.1016/j.febslet.2007.04.054

[57] C. G. Knight, L. F. Morton, A. R. Peachey, D. S. Tuckwell, R. W. Farndale, M. J. Barnes, The collagen-binding A-domains of integrins α_1_β_1_ and α_2_β_1_ recognize the same specific amino acid sequence, GFOGER, in native (triple-helical) collagens. J. Biol. Chem. 275, 35–40 (2000). 10617582 10.1074/jbc.275.1.35

[58] Y. Zou, D. Zwolanek, Y. Izu, S. Gandhy, G. Schreiber, K. Brockmann, M. Devoto, Z. Tian, Y. Hu, G. Veit, M. Meier, J. Stetefeld, D. Hicks, V. Straub, N. C. Voermans, D. E. Birk, E. R. Barton, M. Koch, C. G. Bönnemann, Recessive and dominant mutations in *COL12A1* cause a novel EDS/myopathy overlap syndrome in humans and mice. Hum. Mol. Genet. 23, 2339–2352 (2014). 24334604 10.1093/hmg/ddt627 PMC3976332

[59] R.-Z. Zhang, Y. Zou, T.-C. Pan, D. Markova, A. Fertala, Y. Hu, S. Squarzoni, U. C. Reed, S. K. N. Marie, C. G. Bönnemann, M.-L. Chu, Recessive COL6A2 C-globular missense mutations in Ullrich congenital muscular dystrophy: Role of thE C2a splice variant. J. Biol. Chem. 285, 10005–10015 (2010). 20106987 10.1074/jbc.M109.093666 PMC2843164

[60] A. Irie, T. Kamata, W. Puzon-McLaughlin, Y. Takada, Critical amino acid residues for ligand binding are clustered in a predicted beta-turn of the third N-terminal repeat in the integrin alpha 4 and alpha 5 subunits. EMBO J. 14, 5550–5556 (1995). 8521812 10.1002/j.1460-2075.1995.tb00242.x PMC394669

[61] M. Sen, T. A. Springer, Leukocyte integrin α_L_β_2_ headpiece structures: The αI domain, the pocket for the internal ligand, and concerted movements of its loops. Proc. Natl. Acad. Sci. U.S.A. 113, 2940–2945 (2016). 26936951 10.1073/pnas.1601379113 PMC4801256

[62] B. S. Chouhan, J. Käpylä, K. Denessiouk, A. Denesyuk, J. Heino, M. S. Johnson, Early chordate origin of the vertebrate integrin αI domains. PLOS ONE 9, e112064 (2014). 25409021 10.1371/journal.pone.0112064 PMC4237329

[63] G. Apic, J. Gough, S. A. Teichmann, Domain combinations in archaeal, eubacterial and eukaryotic proteomes. J. Mol. Biol. 310, 311–325 (2001). 11428892 10.1006/jmbi.2001.4776

[64] O. J. Harrison, F. Bahna, P. S. Katsamba, X. Jin, J. Brasch, J. Vendome, G. Ahlsen, K. J. Carroll, S. R. Price, B. Honig, L. Shapiro, Two-step adhesive binding by classical cadherins. Nat. Struct. Mol. Biol. 17, 348–357 (2010). 20190754 10.1038/nsmb.1784 PMC2872554

[65] M. Ohi, Y. Li, Y. Cheng, T. Walz, Negative staining and image classification — Powerful tools in modern electron microscopy. Biol. Proced. Online 6, 23–34 (2004). 15103397 10.1251/bpo70 PMC389902

[66] C. Suloway, J. Pulokas, D. Fellmann, A. Cheng, F. Guerra, J. Quispe, S. Stagg, C. S. Potter, B. Carragher, Automated molecular microscopy: The new Leginon system. J. Struct. Biol. 151, 41–60 (2005). 15890530 10.1016/j.jsb.2005.03.010

[67] A. Punjani, J. L. Rubinstein, D. J. Fleet, M. A. Brubaker, cryoSPARC: Algorithms for rapid unsupervised cryo-EM structure determination. Nat. Methods 14, 290–296 (2017). 28165473 10.1038/nmeth.4169

[68] M. J. Humphries, Integrin activation: The link between ligand binding and signal transduction. Curr. Opin. Cell Biol. 8, 632–640 (1996). 8939662 10.1016/s0955-0674(96)80104-9

[69] I. Dransfield, C. Cabañas, A. Craig, N. Hogg, Divalent cation regulation of the function of the leukocyte integrin LFA-1. J. Cell Biol. 116, 219–226 (1992). 1346139 10.1083/jcb.116.1.219 PMC2289255

[70] D. N. Mastronarde, Automated electron microscope tomography using robust prediction of specimen movements. J. Struct. Biol. 152, 36–51 (2005). 16182563 10.1016/j.jsb.2005.07.007

[71] S. Q. Zheng, E. Palovcak, J.-P. Armache, K. A. Verba, Y. Cheng, D. A. Agard, MotionCor2: Anisotropic correction of beam-induced motion for improved cryo-electron microscopy. Nat. Methods 14, 331–332 (2017). 28250466 10.1038/nmeth.4193 PMC5494038

[72] J. Zivanov, T. Nakane, B. O. Forsberg, D. Kimanius, W. J. H. Hagen, E. Lindahl, S. H. W. Scheres, New tools for automated high-resolution cryo-EM structure determination in RELION-3. eLife 7, e42166 (2018). 30412051 10.7554/eLife.42166 PMC6250425

[73] E. F. Pettersen, T. D. Goddard, C. C. Huang, E. C. Meng, G. S. Couch, T. I. Croll, J. H. Morris, T. E. Ferrin, UCSF ChimeraX: Structure visualization for researchers, educators, and developers. Protein Sci. 30, 70–82 (2021). 32881101 10.1002/pro.3943 PMC7737788

[74] M. A. Cianfrocco, M. Wong-Barnum, C. Youn, R. Wagner, A. Leschziner, “COSMIC2: A Science gateway for cryo-electron microscopy structure determination,” in *Proceedings of the Practice and Experience in Advanced Research Computing 2017 on Sustainability, Success and Impact* (Association for Computing Machinery, 2017), pp. 1–5.

[75] R. Sanchez-Garcia, J. Gomez-Blanco, A. Cuervo, J. M. Carazo, C. O. S. Sorzano, J. Vargas, DeepEMhancer: A deep learning solution for cryo-EM volume post-processing. Commun. Biol. 4, 874 (2021). 34267316 10.1038/s42003-021-02399-1 PMC8282847

[76] Y. Z. Tan, P. R. Baldwin, J. H. Davis, J. R. Williamson, C. S. Potter, B. Carragher, D. Lyumkis, Addressing preferred specimen orientation in single-particle cryo-EM through tilting. Nat. Methods 14, 793–796 (2017). 28671674 10.1038/nmeth.4347 PMC5533649

[77] A. Punjani, D. J. Fleet, 3DFlex: Determining structure and motion of flexible proteins from cryo-EM. Nat. Methods 20, 860–870 (2023). 37169929 10.1038/s41592-023-01853-8 PMC10250194

[78] K. Jamali, L. Käll, R. Zhang, A. Brown, D. Kimanius, S. H. W. Scheres, Automated model building and protein identification in cryo-EM maps. Nature 628, 450–457 (2024). 38408488 10.1038/s41586-024-07215-4 PMC11006616

[79] P. D. Adams, P. V. Afonine, G. Bunkóczi, V. B. Chen, I. W. Davis, N. Echols, J. J. Headd, L.-W. Hung, G. J. Kapral, R. W. Grosse-Kunstleve, A. J. McCoy, N. W. Moriarty, R. Oeffner, R. J. Read, D. C. Richardson, J. S. Richardson, T. C. Terwilliger, P. H. Zwart, PHENIX: A comprehensive Python-based system for macromolecular structure solution. Acta Crystallogr. D Biol. Crystallogr. 66, 213–221 (2010). 20124702 10.1107/S0907444909052925 PMC2815670

[80] A. Waterhouse, M. Bertoni, S. Bienert, G. Studer, G. Tauriello, R. Gumienny, F. T. Heer, T. A. P. de Beer, C. Rempfer, L. Bordoli, R. Lepore, T. Schwede, SWISS-MODEL: Homology modelling of protein structures and complexes. Nucleic Acids Res. 46, W296–W303 (2018). 29788355 10.1093/nar/gky427 PMC6030848

[81] V. Nardone, A. P. Lucarelli, A. Dalle Vedove, R. Fanelli, A. Tomassetti, L. Belvisi, M. Civera, E. Parisini, Crystal structure of human E-cadherin-EC1EC2 in complex with a peptidomimetic competitive inhibitor of cadherin homophilic interaction. J. Med. Chem. 59, 5089–5094 (2016). 27120112 10.1021/acs.jmedchem.5b01487

[82] R. Evans, M. O’Neill, A. Pritzel, N. Antropova, A. Senior, T. Green, A. Žídek, R. Bates, S. Blackwell, J. Yim, O. Ronneberger, S. Bodenstein, M. Zielinski, A. Bridgland, A. Potapenko, A. Cowie, K. Tunyasuvunakool, R. Jain, E. Clancy, P. Kohli, J. Jumper, D. Hassabis, Protein complex prediction with AlphaFold-Multimer. bioRxiv 2021.10.04.463034 [Preprint] (2022). 10.1101/2021.10.04.463034.

[83] P. Emsley, K. Cowtan, Coot: Model-building tools for molecular graphics. Acta Crystallogr. D Biol. Crystallogr. 60, 2126–2132 (2004). 15572765 10.1107/S0907444904019158

[84] T. I. Croll, ISOLDE: A physically realistic environment for model building into low-resolution electron-density maps. Acta Crystallogr. D Struct. Biol. 74, 519–530 (2018). 29872003 10.1107/S2059798318002425 PMC6096486

[85] A. Leaver-Fay, M. Tyka, S. M. Lewis, O. F. Lange, J. Thompson, R. Jacak, K. Kaufman, P. D. Renfrew, C. A. Smith, W. Sheffler, I. W. Davis, S. Cooper, A. Treuille, D. J. Mandell, F. Richter, Y.-E. A. Ban, S. J. Fleishman, J. E. Corn, D. E. Kim, S. Lyskov, M. Berrondo, S. Mentzer, Z. Popović, J. J. Havranek, J. Karanicolas, R. Das, J. Meiler, T. Kortemme, J. J. Gray, B. Kuhlman, D. Baker, P. Bradley, ROSETTA3: An object-oriented software suite for the simulation and design of macromolecules. Methods Enzymol. 487, 545–574 (2011). 21187238 10.1016/B978-0-12-381270-4.00019-6 PMC4083816

[86] E. Krissinel, K. Henrick, Inference of macromolecular assemblies from crystalline state. J. Mol. Biol. 372, 774–797 (2007). 17681537 10.1016/j.jmb.2007.05.022

[87] M. Jin, R. I. Seed, G. Cai, T. Shing, L. Wang, S. Ito, A. Cormier, S. A. Wankowicz, J. M. Jespersen, J. L. Baron, N. D. Carey, M. G. Campbell, Z. Yu, P. K. Tang, P. Cossio, W. Wen, J. Lou, J. Marks, S. L. Nishimura, Y. Cheng, Dynamic allostery drives autocrine and paracrine TGF-β signaling. Cell 187, 6200–6219.e23 (2024). 39288764 10.1016/j.cell.2024.08.036 PMC11531391

[88] H. Zhang, N. S. Astrof, J.-H. Liu, J.-H. Wang, M. Shimaoka, Crystal structure of isoflurane bound to integrin LFA-1 supports a unified mechanism of volatile anesthetic action in the immune and central nervous systems. FASEB J. 23, 2735–2740 (2009). 19332643 10.1096/fj.09-129908 PMC2717780

[89] K. L. Brown, S. Banerjee, A. Feigley, H. Abe, T. S. Blackwell, A. Pozzi, B. G. Hudson, R. Zent, Salt-bridge modulates differential calcium-mediated ligand binding to integrin α1- and α2-I domains. Sci. Rep. 8, 1–14 (2018). 29440721 10.1038/s41598-018-21231-1 PMC5811549

[90] T. Keren-Kaplan, L. Zeev Peters, O. Levin-Kravets, I. Attali, O. Kleifeld, N. Shohat, S. Artzi, O. Zucker, I. Pilzer, N. Reis, M. H. Glickman, S. Ben-Aroya, G. Prag, Structure of ubiquitylated-Rpn10 provides insight into its autoregulation mechanism. Nat. Commun. 7, 12960 (2016). 27698474 10.1038/ncomms12960 PMC5059453

[91] J. Abramson, J. Adler, J. Dunger, R. Evans, T. Green, A. Pritzel, O. Ronneberger, L. Willmore, A. J. Ballard, J. Bambrick, S. W. Bodenstein, D. A. Evans, C.-C. Hung, M. O’Neill, D. Reiman, K. Tunyasuvunakool, Z. Wu, A. Žemgulytė, E. Arvaniti, C. Beattie, O. Bertolli, A. Bridgland, A. Cherepanov, M. Congreve, A. I. Cowen-Rivers, A. Cowie, M. Figurnov, F. B. Fuchs, H. Gladman, R. Jain, Y. A. Khan, C. M. R. Low, K. Perlin, A. Potapenko, P. Savy, S. Singh, A. Stecula, A. Thillaisundaram, C. Tong, S. Yakneen, E. D. Zhong, M. Zielinski, A. Žídek, V. Bapst, P. Kohli, M. Jaderberg, D. Hassabis, J. M. Jumper, Accurate structure prediction of biomolecular interactions with AlphaFold 3. Nature 630, 493–500 (2024). 38718835 10.1038/s41586-024-07487-w PMC11168924

[92] M. Mirdita, K. Schütze, Y. Moriwaki, L. Heo, S. Ovchinnikov, M. Steinegger, ColabFold: Making protein folding accessible to all. Nat. Methods 19, 679–682 (2022). 35637307 10.1038/s41592-022-01488-1 PMC9184281

[93] P. Eastman, J. Swails, J. D. Chodera, R. T. McGibbon, Y. Zhao, K. A. Beauchamp, L.-P. Wang, A. C. Simmonett, M. P. Harrigan, C. D. Stern, R. P. Wiewiora, B. R. Brooks, V. S. Pande, OpenMM 7: Rapid development of high performance algorithms for molecular dynamics. PLOS Comput. Biol. 13, e1005659 (2017). 28746339 10.1371/journal.pcbi.1005659 PMC5549999

[94] P. Eastman, R. Galvelis, R. P. Peláez, C. R. A. Abreu, S. E. Farr, E. Gallicchio, A. Gorenko, M. M. Henry, F. Hu, J. Huang, A. Krämer, J. Michel, J. A. Mitchell, V. S. Pande, J. P. Rodrigues, J. Rodriguez-Guerra, A. C. Simmonett, S. Singh, J. Swails, P. Turner, Y. Wang, I. Zhang, J. D. Chodera, G. De Fabritiis, T. E. Markland, OpenMM 8: Molecular dynamics simulation with machine learning potentials. J. Phys. Chem. B 128, 109–116 (2024). 38154096 10.1021/acs.jpcb.3c06662 PMC10846090

[95] C. Tian, K. Kasavajhala, K. A. A. Belfon, L. Raguette, H. Huang, A. N. Migues, J. Bickel, Y. Wang, J. Pincay, Q. Wu, C. Simmerling, ff19SB: Amino-acid-specific protein backbone parameters trained against quantum mechanics energy surfaces in solution. J. Chem. Theory Comput. 16, 528–552 (2020). 31714766 10.1021/acs.jctc.9b00591 PMC13071887

[96] A. Sengupta, Z. Li, L. F. Song, P. Li, K. M. Merz Jr., Parameterization of monovalent ions for the OPC3, OPC, TIP3P-FB, and TIP4P-FB water models. J. Chem. Inf. Model. 61, 869–880 (2021). 33538599 10.1021/acs.jcim.0c01390 PMC8173365

[97] C. W. Hopkins, S. Le Grand, R. C. Walker, A. E. Roitberg, Long-time-step molecular dynamics through hydrogen mass repartitioning. J. Chem. Theory Comput. 11, 1864–1874 (2015). 26574392 10.1021/ct5010406

[98] D. R. Roe, T. E. Cheatham III, PTRAJ and CPPTRAJ: Software for processing and analysis of molecular dynamics trajectory data. J. Chem. Theory Comput. 9, 3084–3095 (2013). 26583988 10.1021/ct400341p

[99] R. T. McGibbon, K. A. Beauchamp, M. P. Harrigan, C. Klein, J. M. Swails, C. X. Hernández, C. R. Schwantes, L.-P. Wang, T. J. Lane, V. S. Pande, MDTraj: A modern open library for the analysis of molecular dynamics trajectories. Biophys. J. 109, 1528–1532 (2015). 26488642 10.1016/j.bpj.2015.08.015 PMC4623899

[100] L. McInnes, J. Healy, J. Melville, UMAP: Uniform manifold approximation and projection for dimension reduction. arXiv:1802.03426 [stat.ML] (2018).

[101] R. D. Finn, J. Clements, S. R. Eddy, HMMER web server: Interactive sequence similarity searching. Nucleic Acids Res. 39, W29–W37 (2011). 21593126 10.1093/nar/gkr367 PMC3125773

[102] R. C. Edgar, MUSCLE: A multiple sequence alignment method with reduced time and space complexity. BMC Bioinformatics 5, 113 (2004). 15318951 10.1186/1471-2105-5-113 PMC517706

[103] B. Q. Minh, H. A. Schmidt, O. Chernomor, D. Schrempf, M. D. Woodhams, A. von Haeseler, R. Lanfear, IQ-TREE 2: New models and efficient methods for phylogenetic inference in the genomic era. Mol. Biol. Evol. 37, 1530–1534 (2020). 32011700 10.1093/molbev/msaa015 PMC7182206

[104] A. Rambaut, Figtree: Automatically exported from (Github); https://github.com/rambaut/figtree.

[105] J.-P. Xiong, T. Stehle, R. Zhang, A. Joachimiak, M. Frech, S. L. Goodman, M. A. Arnaout, Crystal structure of the extracellular segment of integrin αVβ3 in complex with an Arg-Gly-Asp ligand. Science 296, 151–155 (2002). 11884718 10.1126/science.1069040

[106] M. G. Campbell, A. Cormier, S. Ito, R. I. Seed, A. J. Bondesson, J. Lou, J. D. Marks, J. L. Baron, Y. Cheng, S. L. Nishimura, Cryo-EM reveals integrin-mediated TGF-β activation without release from latent TGF-β. Cell 180, 490–501.e16 (2020). 31955848 10.1016/j.cell.2019.12.030 PMC7238552

