# Supplementary material for: Molecular exaptation by the integrin αI domain
Source: Sci Adv. 2025 Sep 10;11(37):eadx9567. doi: 10.1126/sciadv.adx9567 (PMC12422189; doi:10.1126/sciadv.adx9567)
Supplement: Supplementary file 1 — Figs. S1 to S13 Tables S1 and S2 Legends for movies S1 to S3 Legends for data S1 to S5 [file sciadv.adx9567_sm.pdf]

Science Advances
AAAS

Supplementary Materials for

Molecular exaptation by the integrin αI domain

Jeremy A. Hollis *et al.*

Corresponding author: Melody G. Campbell, melody@fredhutch.org

Sci. Adv. **11**, eadx9567 (2025)
DOI: 10.1126/sciadv.adx9567

The PDF file includes:

Figs. S1 to S13
Tables S1 and S2
Legends for movies S1 to S3
Legends for data S1 to S5

Other Supplementary Material for this manuscript includes the following:

Movies S1 to S3
Data S1 to S5

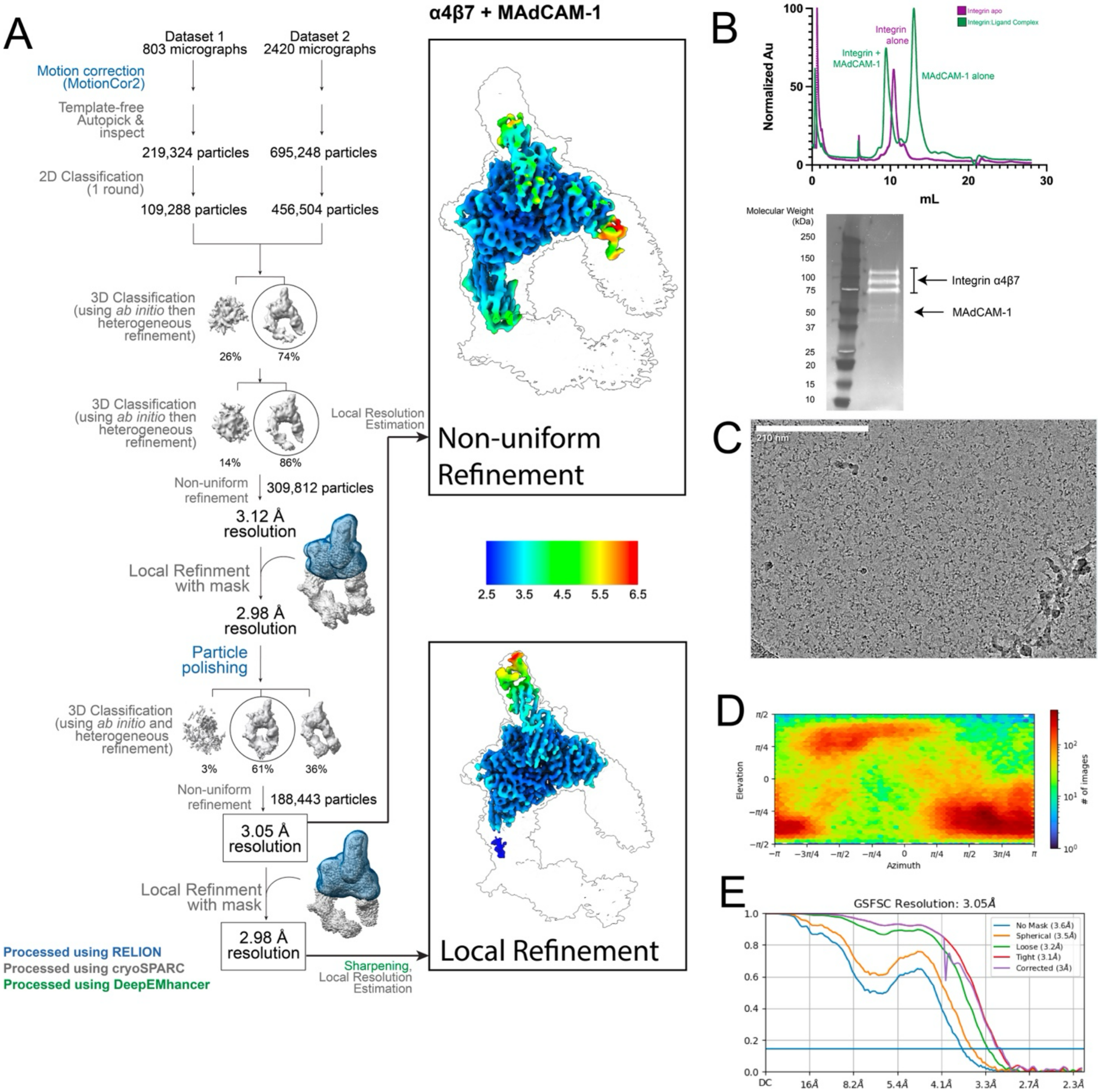

Figure S1. Data processing schematic for the α4β7:MAdCAM-1 complex. (A) A flowchart for the data processing pipeline of α4β7:MAdCAM-1. Both the global and local refinements were used for model building. **(B)** Size exclusion chromatography traces showing peak shift for ligand-bound integrin, with SDS-PAGE of the protein complex fraction shown below. **(C)** Representative micrograph with 210nm scale bar, **(D)** orientational distribution plot, and **(E)** gold-standard Fourier Shell Correlation (GSFSC) plot for the globally refined map.

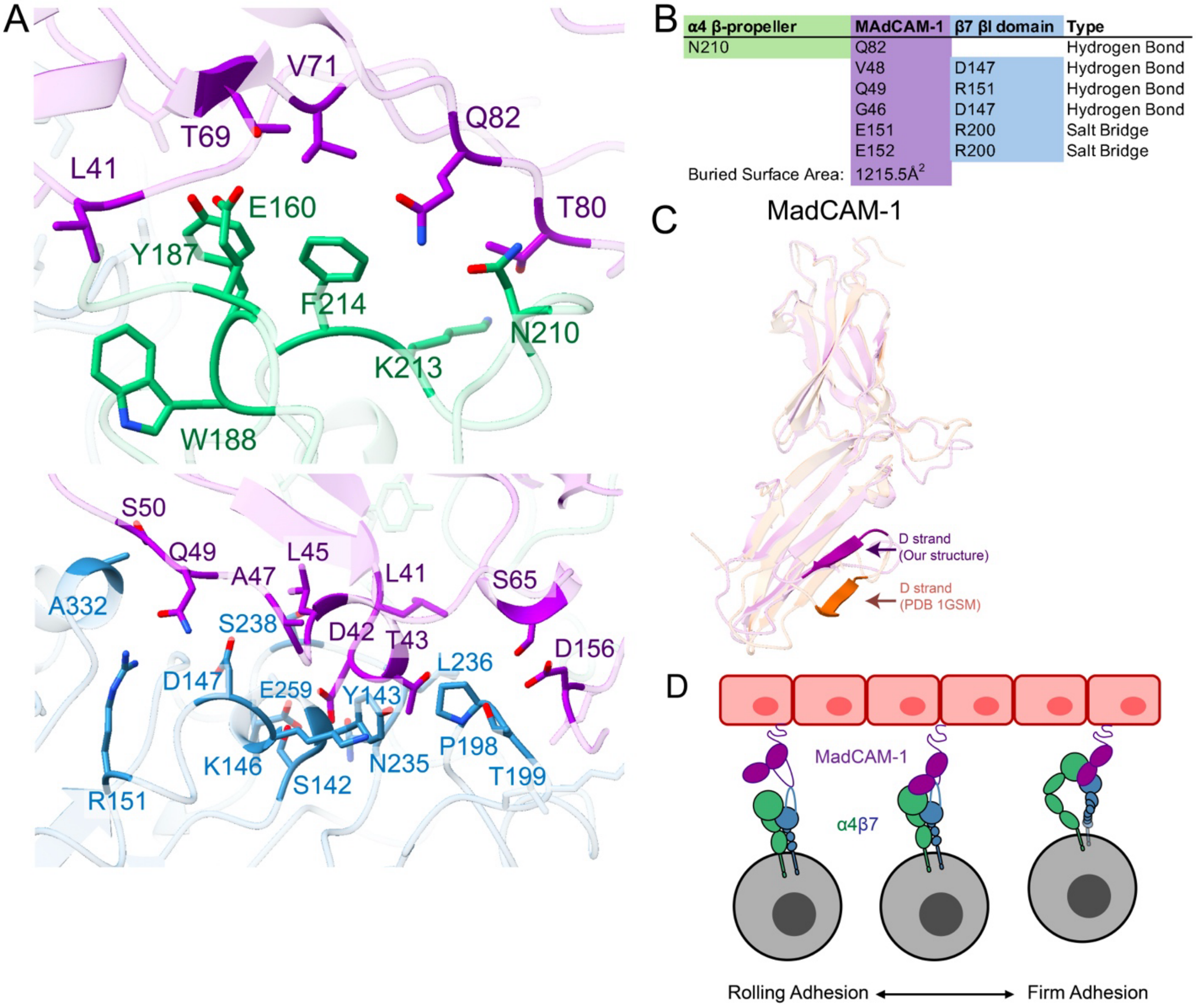

Figure S2. The molecular interface between integrin α4β7 and MAdCAM-1. (A) MAdCAM-1 (purple) binding within the α4β7 groove is stabilized by contacts with both α4 (green, top) and more extensively with β7 (blue, bottom). (**B)** The PISA server was used to determine electrostatic interactions in the α4β7:MAdCAM-1 complex model (**C)** MAdCAM-1 undergoes a conformational shift upon binding to α4β7. The D strand in the first Ig-like domain of MAdCAM-1 is shown in high opacity for both our structure (purple) and the crystal structure (orange, PDB 1GSM). The conformation of the D strand in the crystal structure of MAdCAM-1 alone would sterically clash with our integrin density. **(D)** An integrative model for how α4β7-expressing immune cells structurally identify surveillance sites, slow to roll, and eventually firmly adhere along the gut endothelium.

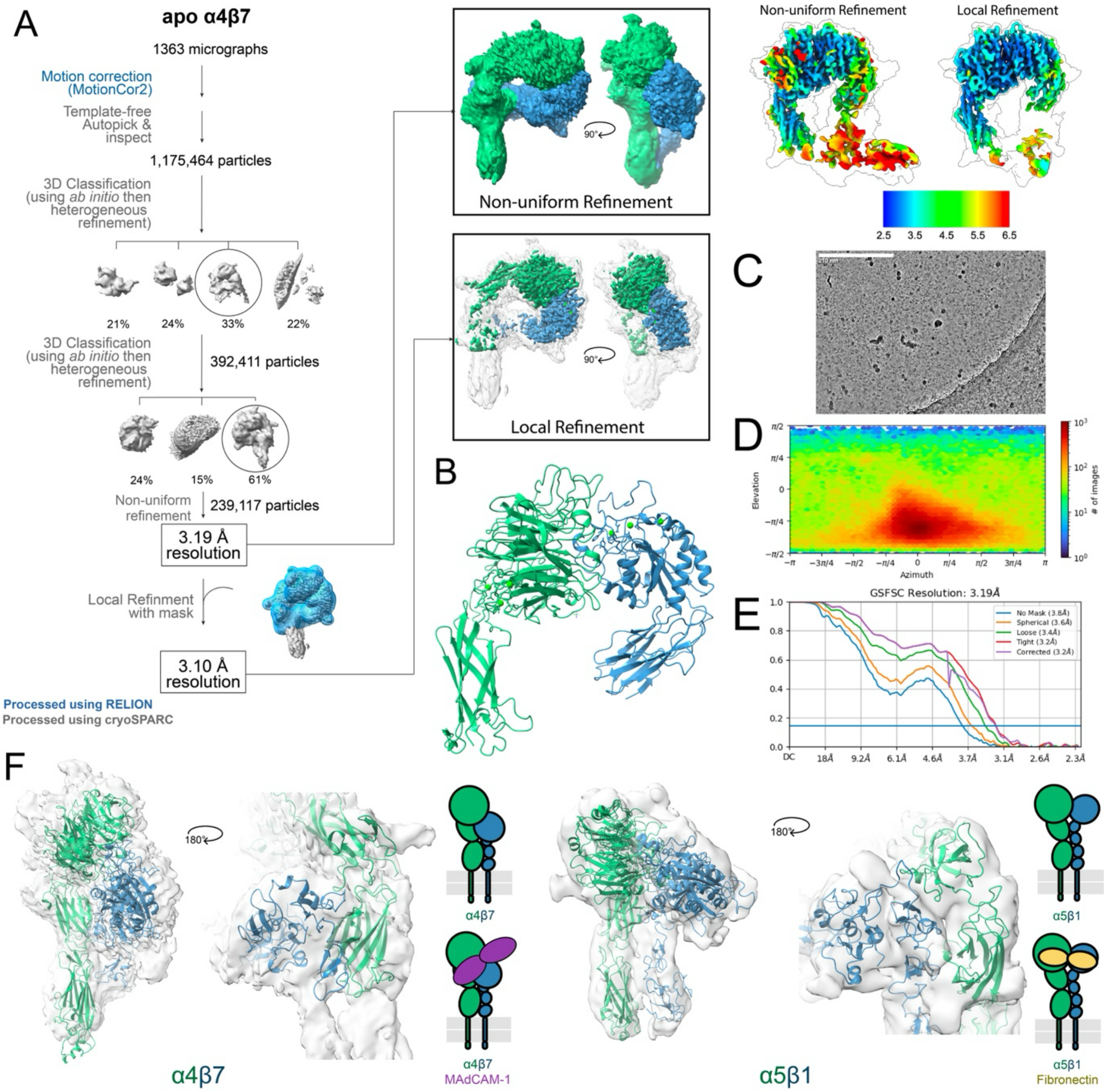

Figure S3. Integrin α4β7 has a novel compact state. (A) A flowchart for the data processing pipeline of apo α4β7. **(B)** The molecular model of the α4β7 headpiece. **(C)** Representative micrograph with 210nm scale bar, **(D)** orientational distribution plot, and **(E)** gold-standard Fourier Shell Correlation (GSFSC) plot for the apo α4β7 structure. **(F)** Unlike integrin α5β1, the compact half-bent state of α4β7 has a significant rotation (40°) at the headpiece mediated by contacts between the two subunits. This may contribute to binding to MAdCAM-1, which binds α4β7 perpendicular compared to how α5β1's ligand fibronectin binds.

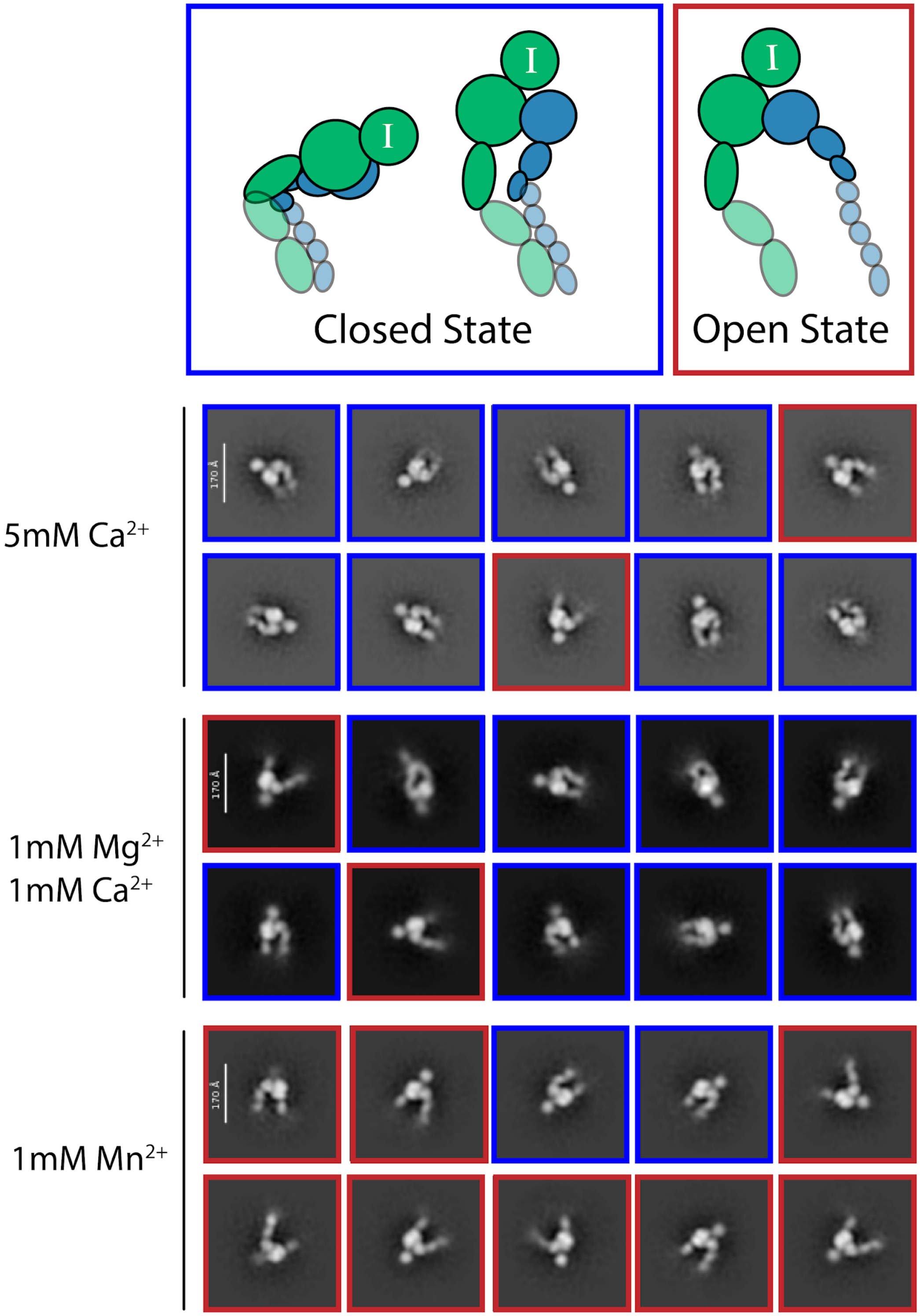

Figure S4. Integrin αEβ7 occupies canonical conformations. Negative stain electron microscopy (nsEM) 2D class averages of integrin αEβ7 in buffers of varying ions show that αEβ7 samples conformations like those previously described for other integrins. The I domain is clearly resolved in each class, however unlike other integrins the lower leg regions remain unclear in negative stain.

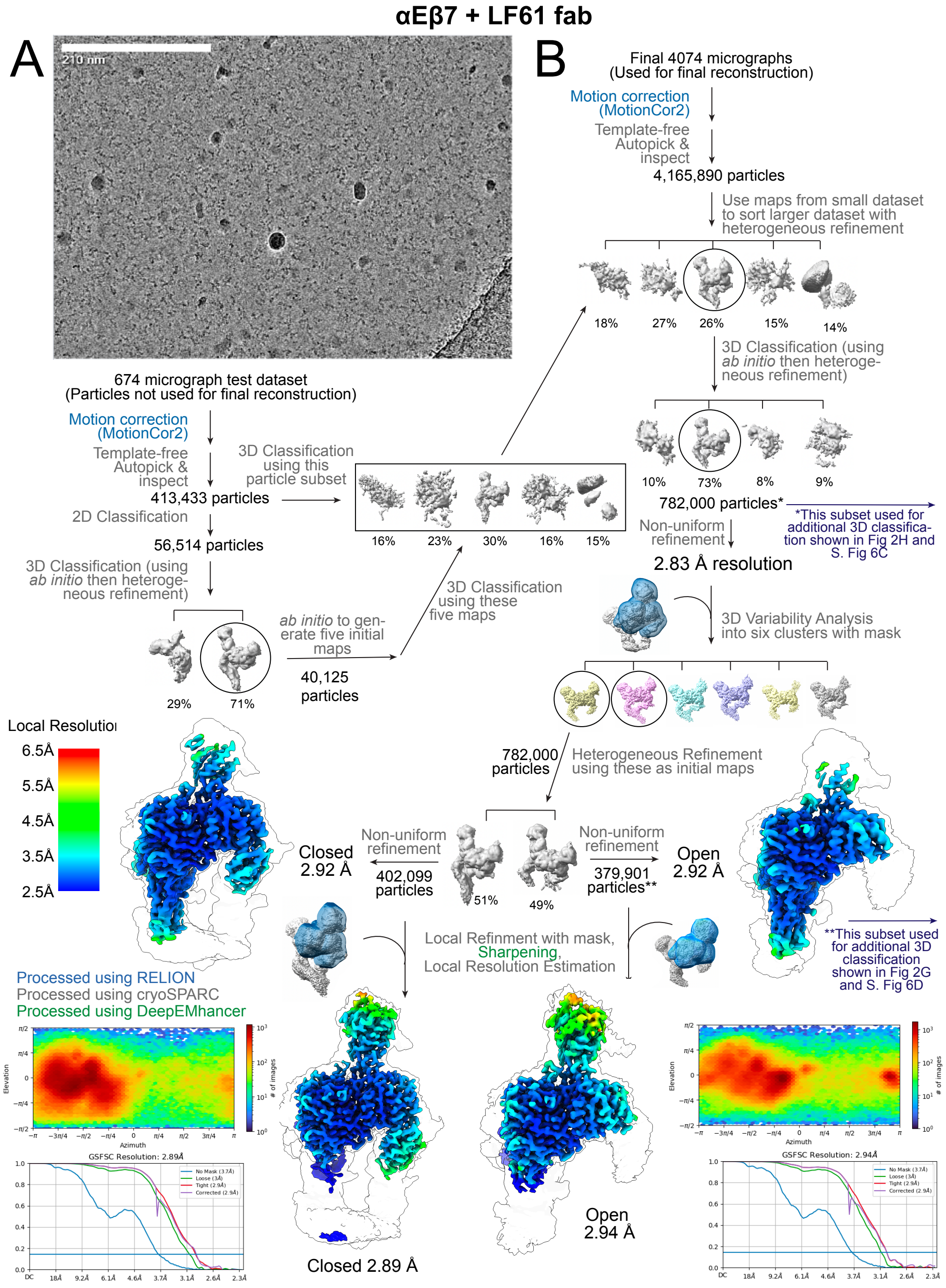

Figure S5. Data processing schematic for the αEβ7:LF61 Fab complex. **(A)** Representative micrograph with 210nm scale bar, and **(B)** a flowchart for the data processing pipeline of αEβ7:LF61 Fab. High-quality particles were sorted into two major classes; those that have an open headpiece and those that had a closed headpiece. We found that the class that has an open headpiece has an open I domain as well and those that have a closed headpiece have a closed I domain. The closed I domain structure, shown on the lower left with local resolution estimates, was used to model the inactive αEβ7 conformation. The open structure is presented in the lower right. Orientational distribution plot and gold-standard Fourier Shell Correlation (GSFSC) plots are shown for each structure.

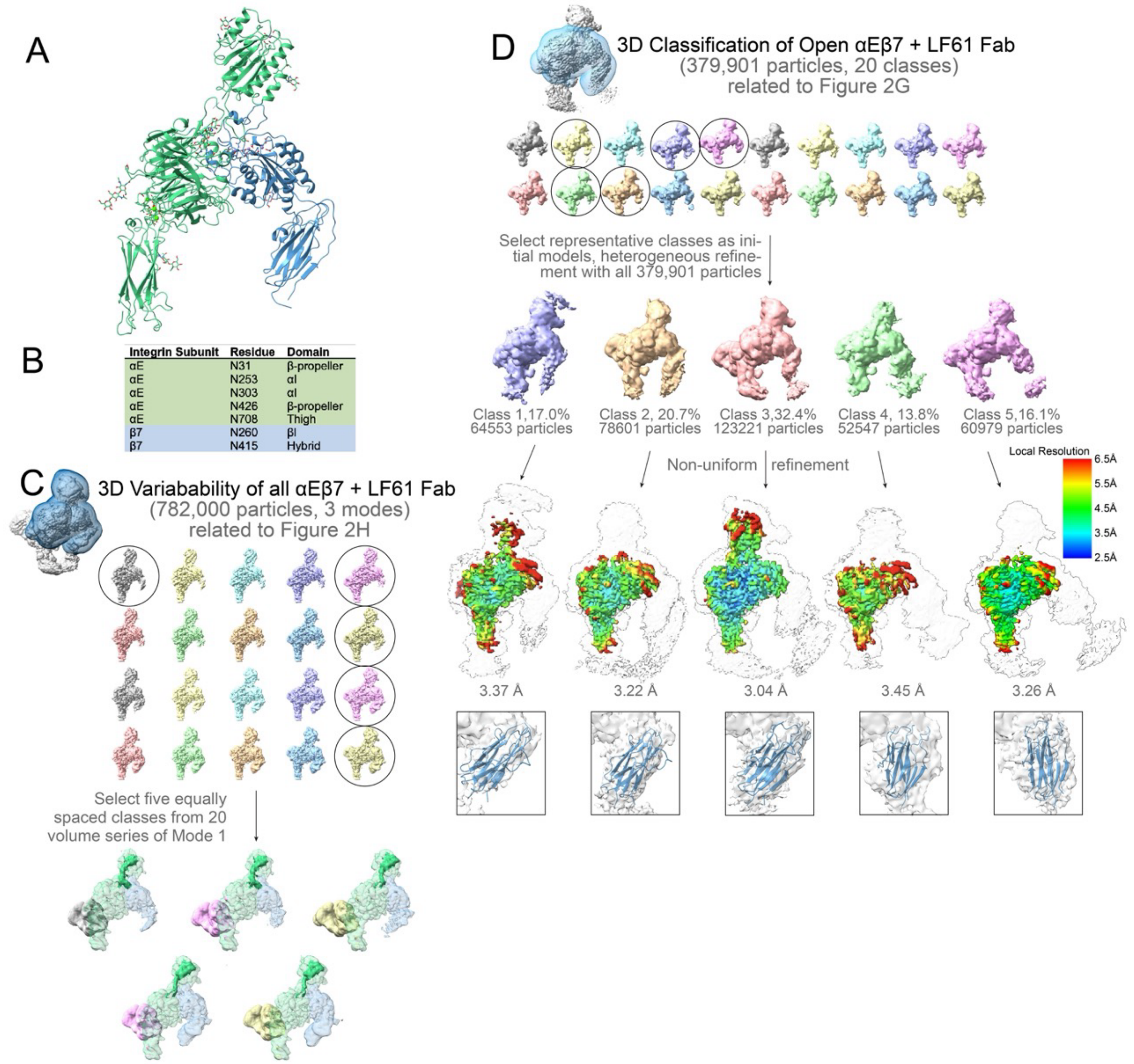

Figure S6. Large-scale and small scale movement characterization of apo αEβ7. (A) The model of the open I domain structure for αEβ7. **(B)** Glycan residues and domains within the αEβ7:LF61 model. **(C)** 3D variability processing schematic for the volume series presented in figure 5H. **(D)** 3D classification to illustrate the large scale movement of the beta-leg relative to the β7 βI domain. The "Open I domain" particles shown in Fig. S5 were subclassified into 20 classes without alignments. Five representative classes with varying β7 leg positions from this classification were used as initial models for a subsequent classification using 3D heterogeneous refinement. Finally, each class was refined to high resolution via non-uniform refinement. The β7 hybrid domain was rigid-fit in each map to calculate leg flexibility.

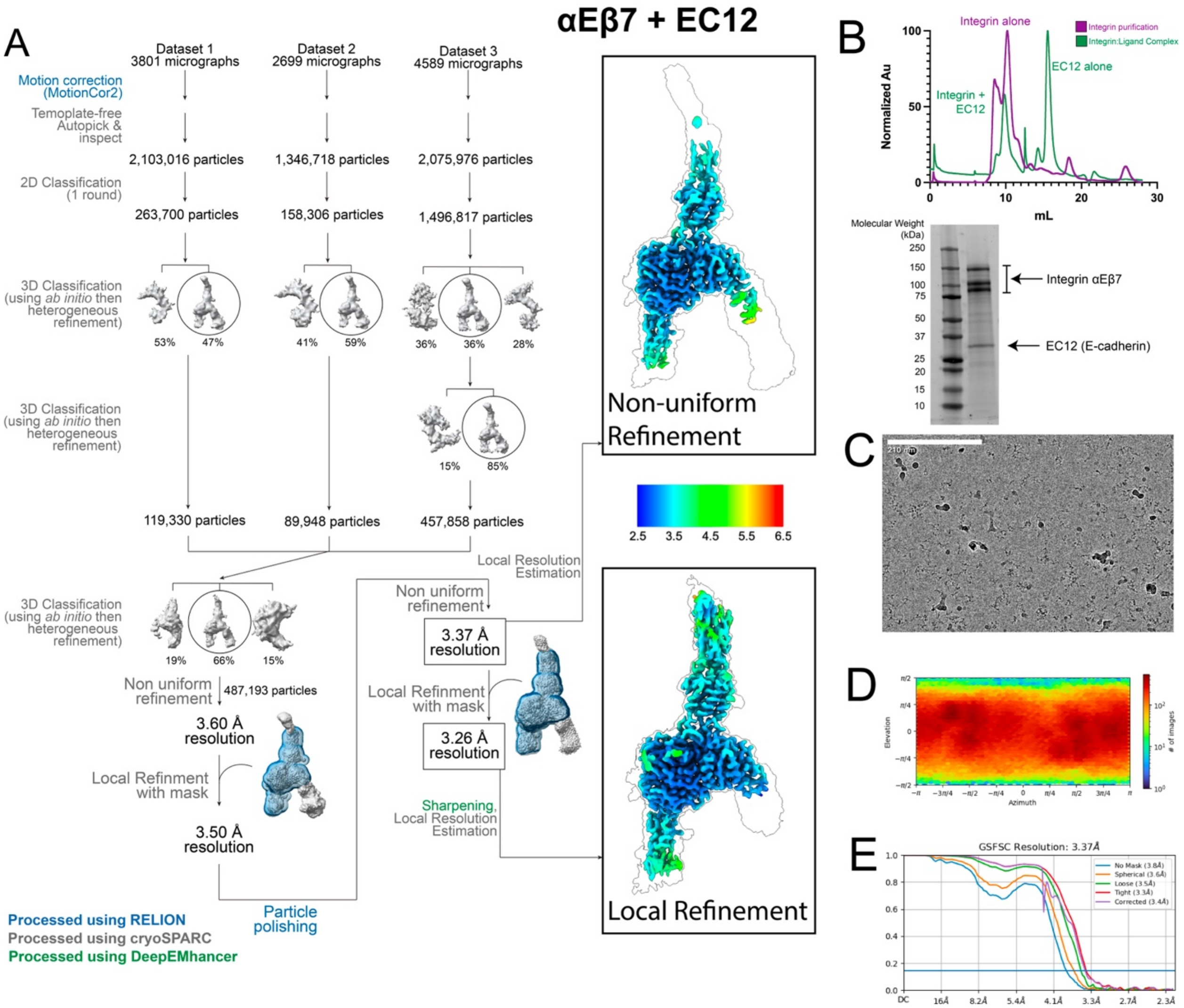

Figure S7. Data processing schematic for the αEβ7:EC12 complex. (A) Flowchart for the data processing pipeline of αEβ7:EC12. Both the global and local refinements were used for model building. **(B)** Size exclusion chromatography traces showing peak shift for ligand-bound integrin, with SDS-PAGE of the protein complex fraction shown below. **(C)** Representative micrograph with 210nm scale bar, **(D)** orientational distribution plot, and **(E)** gold-standard Fourier Shell Correlation (GSFSC) plot of the globally refined map.

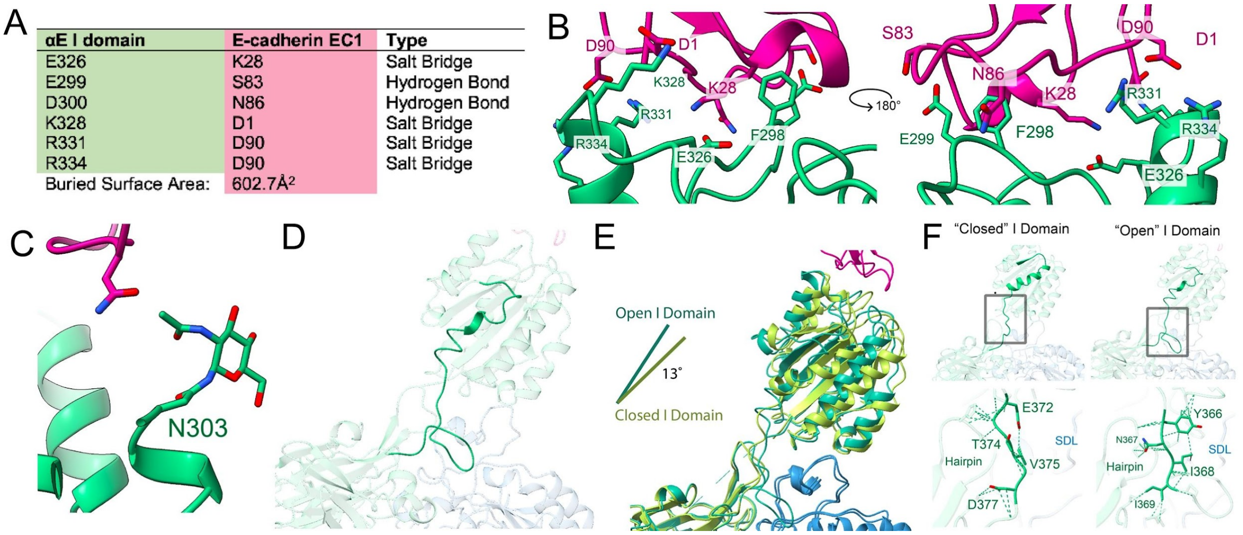

αE I domain	E-cadherin EC1	Type
E326	K28	Salt Bridge
E299	S83	Hydrogen Bond
D300	N86	Hydrogen Bond
K328	D1	Salt Bridge
R331	D90	Salt Bridge
R334	D90	Salt Bridge
Buried Surface Area:	602.7Å^2	

Figure S8. Molecular features of the open integrin αEβ7. (A) Electrostatic interactions in the αEβ7:EC12 complex model between the αE I domain and EC1. **(B)** The interface between αEβ7 and E-cadherin is composed primarily of a central hydrophobic residue, F298, that is surrounded by electrostatic interactions. Views are 180° rotations. **(C)** The N321 glycosylation on the αE I domain contributes to ligand binding. **(D)** The open structure of the αE I domain α7 helix. **(E)** Despite extensive structural changes within the I domain, the location of the I domain relative to the rest of the integrin molecule remains the same when ligand-bound. **(F)** Extensive low-strength contacts stabilize the I domain's internal ligand in either the apo closed state (left) or ligand-bound open state (right).

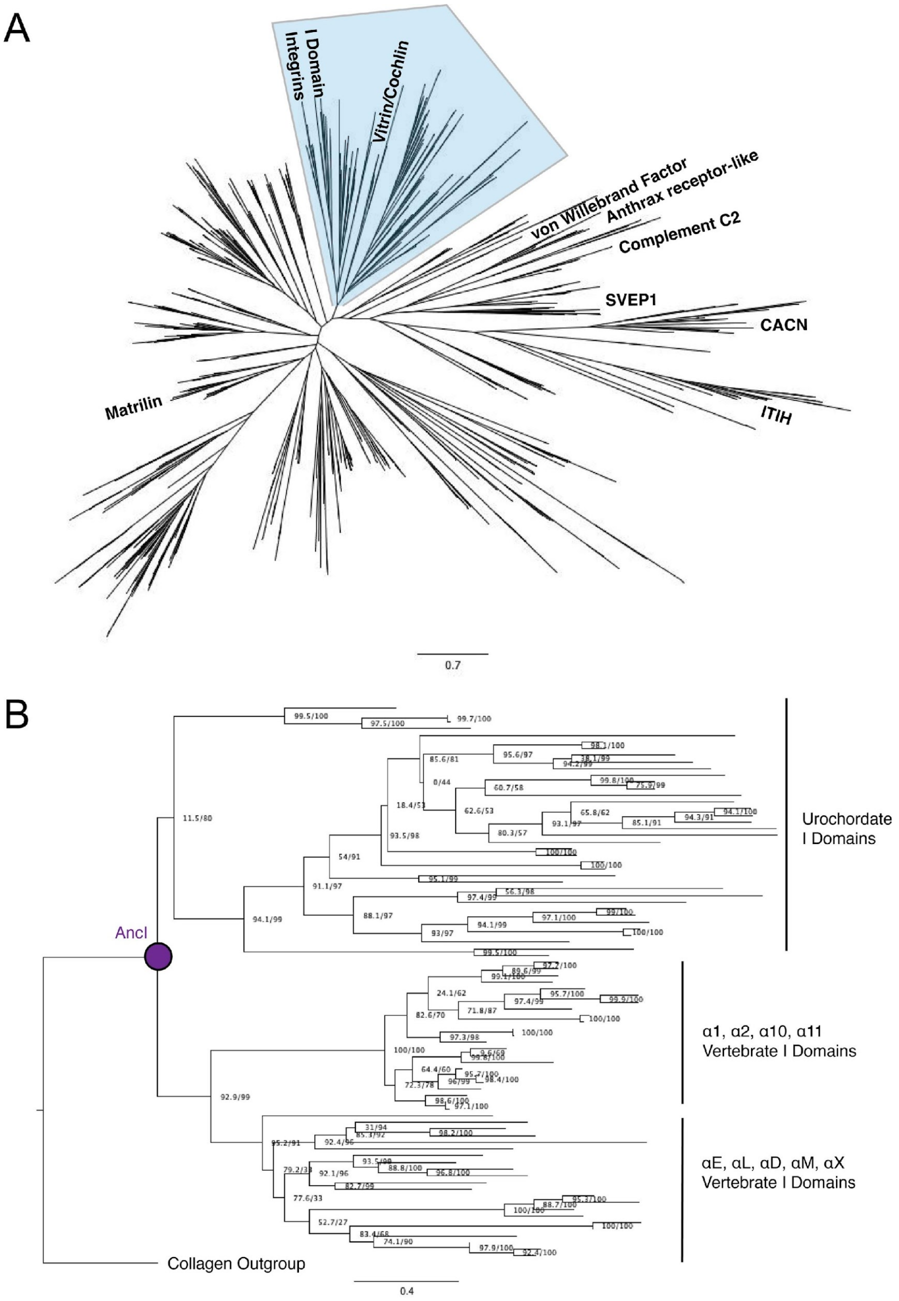

Figure S9. Evolutionary origins of the integrin I domain. (A) A phylogenetic reconstruction of vWFA domains with close homology to the integrin αI domain was generated in IQ-TREE. Blue box indicates subregion of tree presented in Figure 4A. (**B)** A phylogenetic tree of curated I domain sequences used for ancestral sequence reconstruction. UNIPROT C3YQB2 was used as a representative cephalochordate outgroup sequence. Node values are displayed as "ultrafast bootstrap/SH-aLRT".

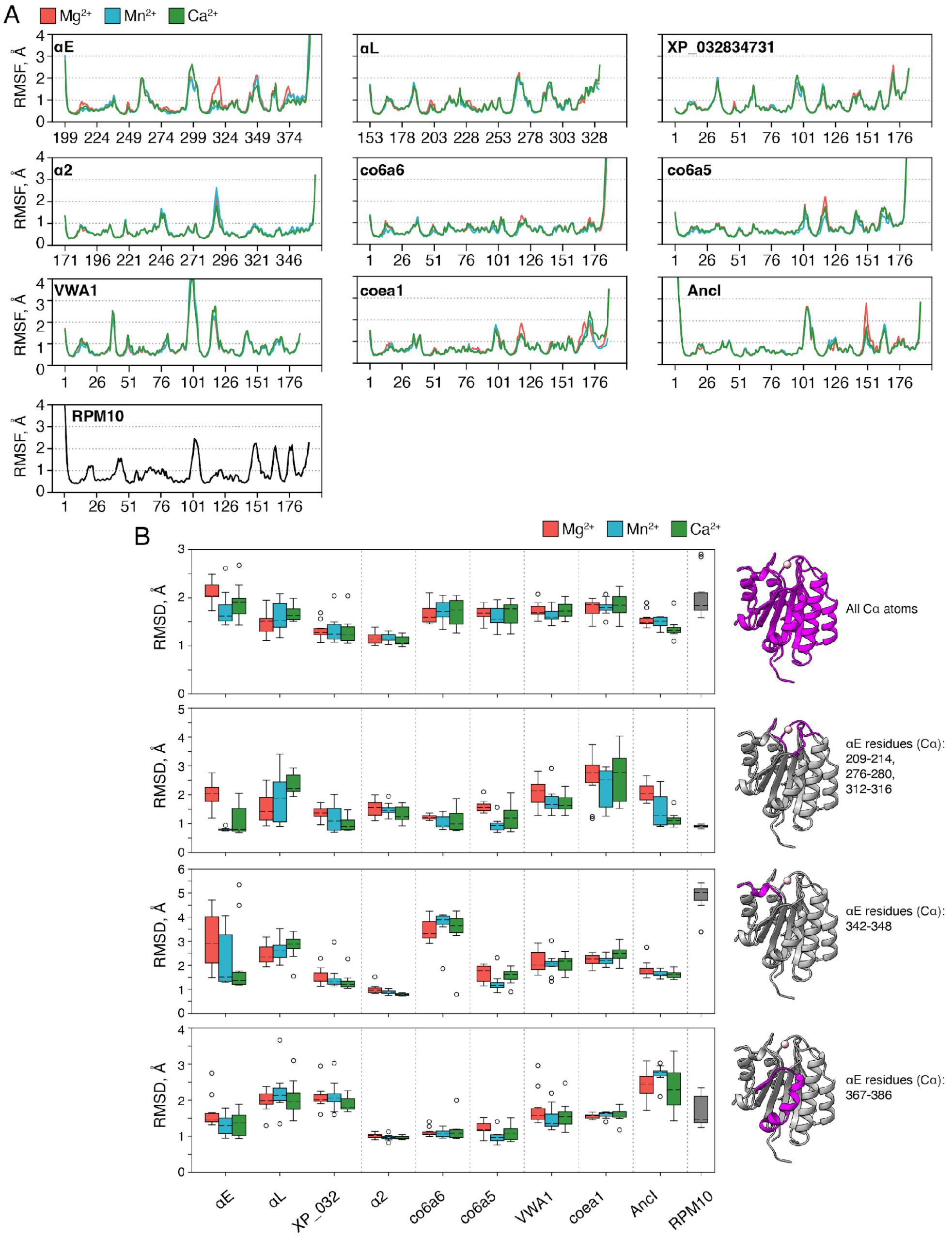

Figure S10. Molecular dynamics simulations reveal conserved dynamics in vWFA domains. (A) Root mean square fluctuation (RMSF) of 10 simulated vWFA proteins with either Mg^{2+} (red), Mn^{2+} (blue), or Ca^{2+} (green) bound in the MIDAS site. For RPN10, no ion was bound. (**B)** RMSD calculation of the simulated vWFA proteins. Color represents the cation bound in the MIDAS site (Mg^{2+}, red; Mn^{2+}, blue; Ca^{2+}, green). Regions calculated, from left to right, all Cα atoms, ion coordinated loops, and sites of conformational change and colored magenta in the structure. Error bars represent standard error. For both (A) and (B), values are averaged across 10 independent replicates of 2.4µs long with only the last 2.0µs used for analysis and the initial simulation structure used as the reference. Initial model sources are found in Table S2.

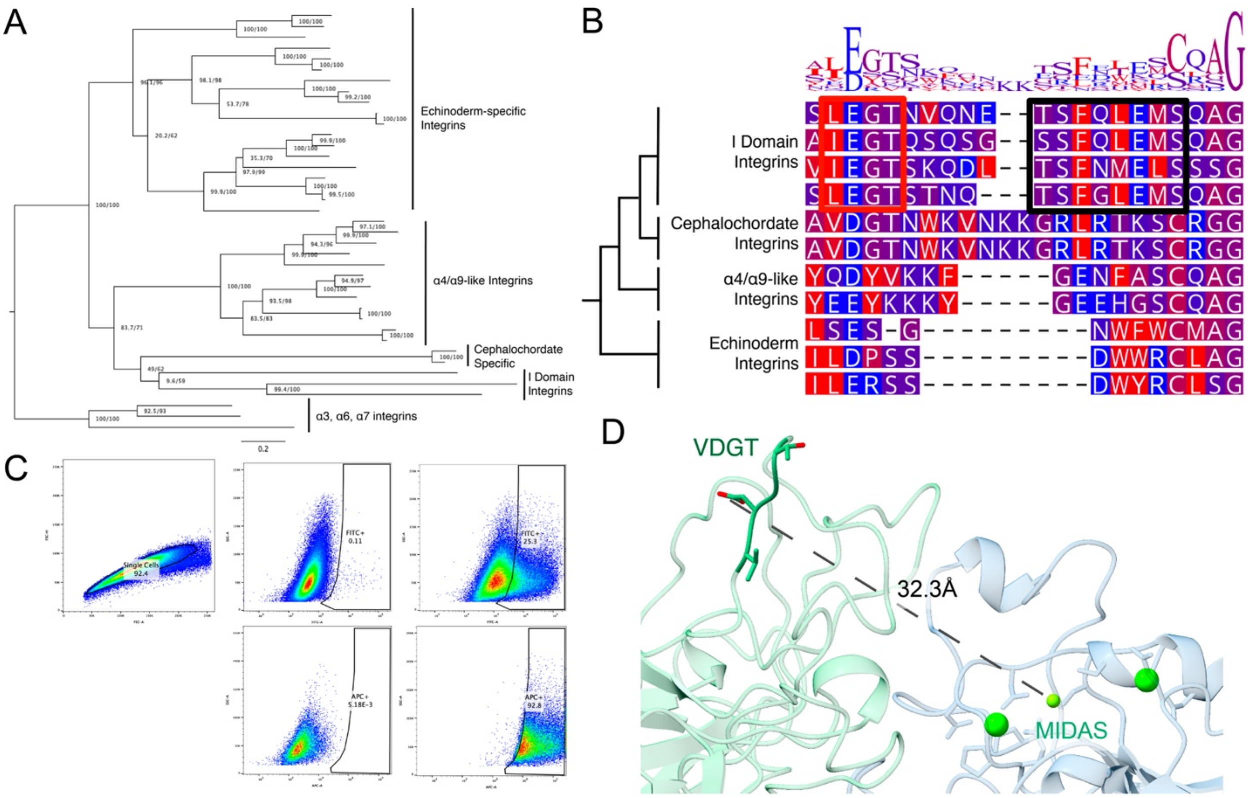

Figure S11. Phylogenetic and structural analyses of the internal ligand origin. (A) Phylogenetic tree of I domain-containing and related integrins. A small clade of genes unique to cephalochordates are the direct sisters of the I domain integrins. Node values are displayed as "ultrafast bootstrap/SH-aLRT". (**B)** Alignment of the loop region between the second and third propellor blade domains of the I domain and related integrins. The direct cephalochordate outgroup contains a VDGT motif sequentially homologous to the I domain internal ligand, while the broader family also encompassing the α4/α9 clade contains a conserved acidic residue akin to the essential ion-coordinating glutamate in the internal ligand. Red square indicates the internal ligand in extant integrins. The black square indicates the structural internal ligand pocket that is largely diverged between I domain integrins and their outgroups. (**C**) Gating strategy used to determine FITC+ and APC+ cells in flow cytometry. Top left: Single cell gating. Top middle: FITC+ gating, negative control. Top right: FITC+ gating, positive control. Bottom middle: APC+ gating, negative control. Bottom right: APC+ gating, positive control. (**D)** Structural homology model of the cephalochordate sister clade I domain-less integrin. The VDGT motif is too distant to the βMIDAS to perform ion coordinating activities, and is thus more likely involved in ligand binding.

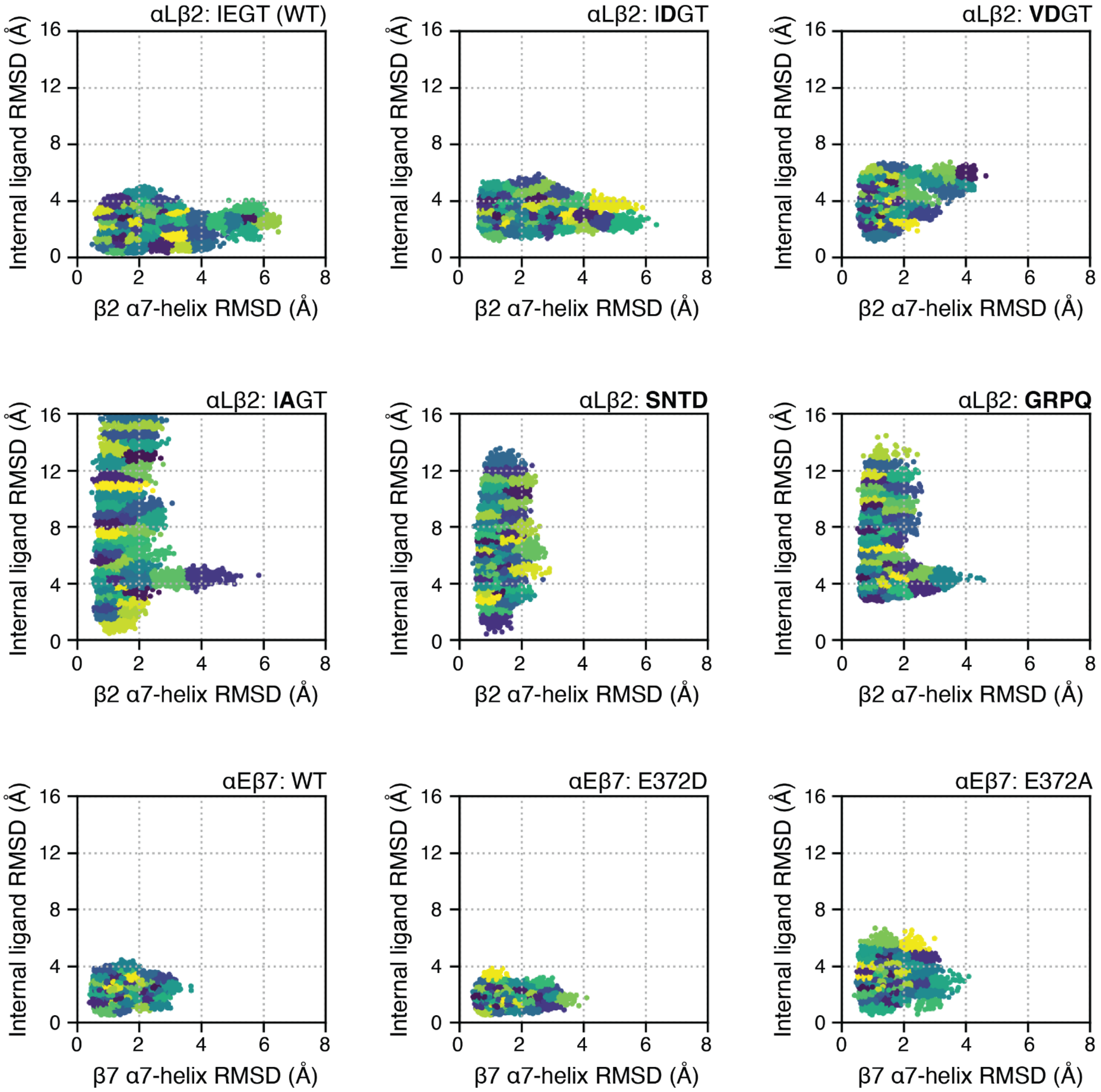

Figure S12. Conformational landscape of αLβ2-ICAM-1 and αEβ7-E-cadherin headpiece domains.

Raw conformational landscape projected on the reaction coordinates of the positional deviation (no-fit RMSD) of the internal ligand and α7 helix of the β subunit clustered in 25 KMeans clusters.

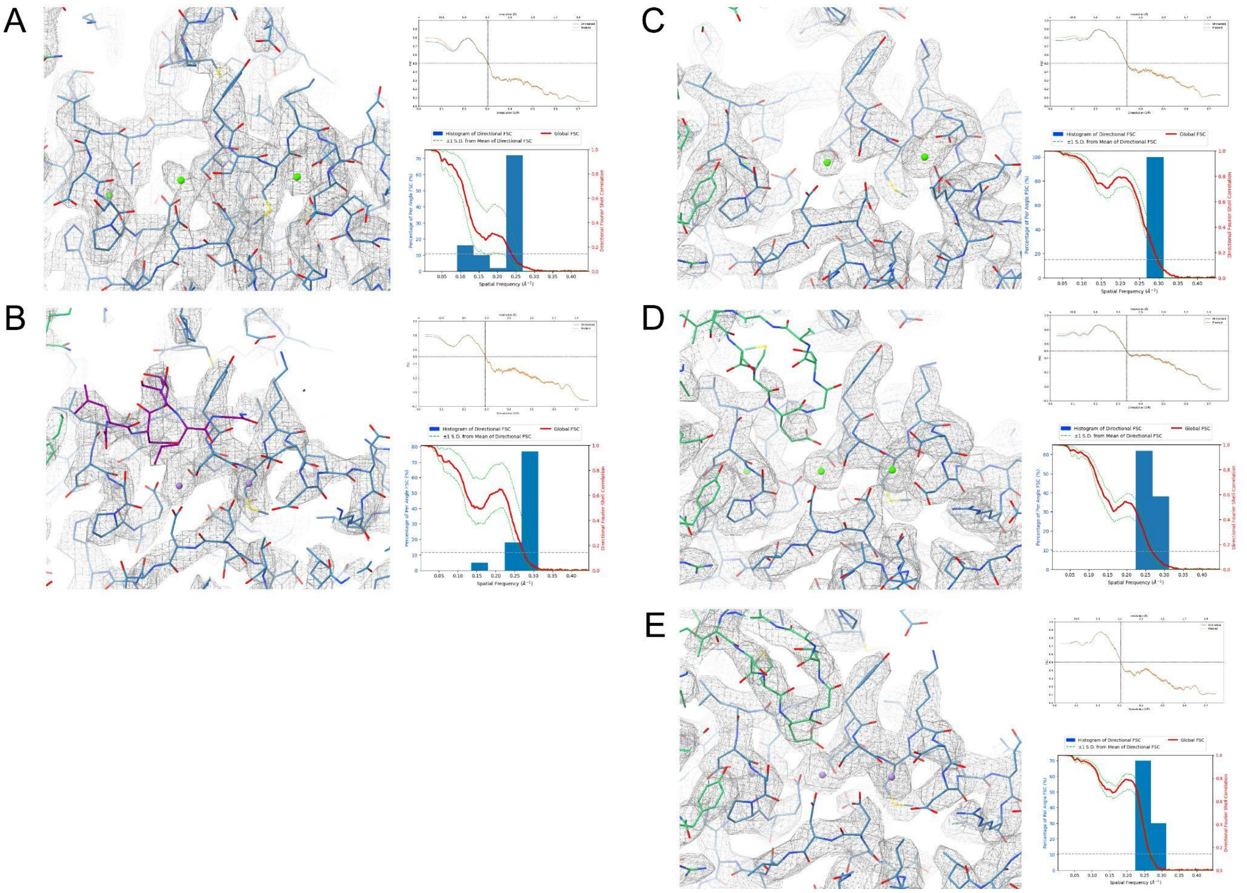

Figure S13. Map quality and validation metrics. Representative map-model fits of the same β7 ion coordination region (left), map-model FSC curves (top right), and 3DFSC curves (bottom right) for **(A)** the apo α4β7, **(B)** the α4β7:MAdCAM-1 complex, **(C)** the αEβ7 apo closed I domain, **(D)** the αEβ7 apo open I domain (as expected, the density for the αE internal ligand is relatively weak at this high threshold), and **(E)** the αEβ7:E-cadherin complex with stronger internal ligand density. In all figures: β subunits are blue; α subunits are green; MAdCAM-1 is purple; E-cadherin is pink.

Table S1. Collection, refinement and validation statistics for cryoEM density maps and models. Collection details for all cryoEM maps presented in the text are shown above, and validation statistics for the models subsequently generated from those maps are shown below.

	αEβ7:LF61 Closed I domain (EMDB 71401) (PDB 9P97)	αEβ7:LF61 Open I domain (EMDB 71402) (PDB 9P98)	αEβ7:EC12 (EMDB 71403) (PDB 9P99)	α4β7:MAdCAM-1 (EMDB 71399) (PDB 9P95)	apo α4β7 (EMDB 71400) (PDB 9P96)
**Data collection and processing**					
Magnification	36000x	36000x	36000x	36000x	36000x
Voltage (kV)	200	200	200	200	200
Electron exposure (e–/Å^2)	50	50	50	50	50
Defocus range (μm, nominal)	1.2-1.8	1.2-1.8	1.2-1.8	1.2-1.8	1.2-1.8
Pixel size (Å)	1.122	1.122	1.122	1.122	1.122
Symmetry imposed	C1	C1	C1	C1	C1
Initial particle images (no.)	4,165,890	4,165,890	5,307,702	914,572	1,175,464
Final particle images (no.)	402,099	379,901	487,193	188,443	202,130
Map resolution (Å)	2.92	2.93	3.37	3.05	3.20
FSC threshold	0.143	0.143	0.143	0.143	0.143
Map resolution range (Å)	2.5-10.2	2.5-16.8	2.8-10.9	2.5-46.5	2.7-47.6
**Refinement**					
Initial model used (Alphafold or PDB code)	Alphafold PDB: 3V4P	αEβ7:EC12	Alphafold PDB: 7NLW, 4ZT1	Alphafold-multimer	Alphafold
Model resolution (Å)	2.92	2.93	3.37	3.05	3.20
FSC threshold	0.143	0.143	0.143	0.143	0.143
Model resolution range (Å)	2.5-10.2	2.5-16.8	2.8-10.9	2.5-46.5	2.7-47.6
Model composition					
Non-hydrogen atoms	8933	8986	9792	9150	7447
Protein residues	1135	1147	1250	1162	960
Carbohydrates	22	18	18	22	0
B factors (Å^2)					
Protein	113.5	71.4	119.7	87.1	66.14
Ligand	N/A	N/A	N/A	N/A	N/A
R.m.s. deviations					
Bond lengths (Å)	0.012 (11)	0.005 (0)	0.012 (12)	0.012 (10)	0.007 (2)
Bond angles (°)	1.949 (52)	0.662 (4)	2.038 (61)	2.048 (73)	0.748 (9)
Validation					
MolProbity score	0.78	1.69	0.82	0.97	2.13
Clashscore	0.17	4.79	0.05	0.22	14.69
Poor rotamers (%)	0.21	1.35	0.09	0.20	0.61
Ramachandran plot					
Favored (%)	96.63	95.00	95.73	94.37	92.68
Allowed (%)	3.19	5.00	3.95	5.55	7.32
Disallowed (%)	0.18	0	0.32	0.09	0.00

Table S2. Molecular dynamics systems simulated in this study. Trajectory lengths and number of replicates of the simulated systems and respectively ligands are shown below.

System	Ligand	Initial Model	Replicates	Trajectory Length (per replicate, μs)
Integrin headpiece				
αEβ7	E-cadherin	This study	4	1.0
αEβ7_E372D	E-cadherin	This study	4	1.0
αEβ7_E372A	E-cadherin	This study	4	1.0
LFA1	ICAM-1	Homology model	6	1.0
LFA1_IDGT	ICAM-1	Homology model	6	1.0
LFA1_VDGT	ICAM-1	Homology model	6	1.0
LFA1_IAGT	ICAM-1	Homology model	6	1.0
LFA1_SNTD	ICAM-1	Homology model	6	1.0
LFA1_GRPQ	ICAM-1	Homology model	6	1.0
I domain				
αE	Mg^{2+}	This study	10	2.4
αE	Mn^{2+}	This study	10	2.4
αE	Ca^{2+}	This study	10	2.4
αL	Mg^{2+}	PDB: 3F74	10	2.4
αL	Mn^{2+}	PDB: 3F74	10	2.4
αL	Ca^{2+}	PDB: 3F74	10	2.4
α2	Mg^{2+}	PDB: 5HJ2	10	2.4
α2	Mn^{2+}	PDB: 5HJ2	10	2.4
α2	Ca^{2+}	PDB: 5HJ2	10	2.4
XP_032834731	Mg^{2+}	Alphafold2	10	2.4
XP_032834731	Mn^{2+}	Alphafold2	10	2.4
XP_032834731	Ca^{2+}	Alphafold2	10	2.4
co6a6	Mg^{2+}	Alphafold3	10	2.4
co6a6	Mn^{2+}	Alphafold3	10	2.4
co6a6	Ca^{2+}	Alphafold3	10	2.4
co6a5	Mg^{2+}	Alphafold3	10	2.4
co6a5	Mn^{2+}	Alphafold3	10	2.4
co6a5	Ca^{2+}	Alphafold3	10	2.4
vwa129	Mg^{2+}	Alphafold3	10	2.4
vwa129	Mn^{2+}	Alphafold3	10	2.4
vwa129	Ca^{2+}	Alphafold3	10	2.4
coea1	Mg^{2+}	Alphafold3	10	2.4
coea1	Mn^{2+}	Alphafold3	10	2.4

coea1	Ca^{2+}	Alphafold3	10	2.4
AncI	Mg^{2+}	Alphafold3	10	2.4
AncI	Mn^{2+}	Alphafold3	10	2.4
AncI	Ca^{2+}	Alphafold3	10	2.4
RPN10	None	PDB: 5LN1	10	2.4

Supplemental Information Legends

Movie S1. Integrin α4β7 is flexible when bound to MAdCAM-1. 3DFlex analysis was used to analyze continuous movement within the α4β7:MAdCAM-1 complex. The β7 subunit (blue) shows hinged motion at the hybrid domain, and there is coordinated rotational movement between the α4 (green) and β7 subunits at the lower leg.

Movie S2. Compact αEβ7 stochastically samples an internally-liganded state. 3D Variability analysis was used to separate compact αEβ7:LF61 particles into states with or without the internal ligand engaged. αE is represented in green and β7 in blue. The internal ligand is the central density with high variability between frames.

Movie S3. Integrin αEβ7 is flexible when bound to E-Cadherin. 3DFlex analysis was used to analyze continuous movement within the αEβ7:EC12 complex. The active β7 subunit (blue) shows a similar hybrid domain motion as in α4β7:MAdCAM-1. There is also some flexible motion between E-cadherin domains EC1 and EC2 (pink). αE is represented in green.

Data S1. Alignment of human α-integrin protein sequences. The alignment used to find the N- and C-terminal I domain insertion regions presented in Fig. 1 in PHYLIP format. Names are presented as gene names.

Data S2. An atomic contact list for the αE I domain:E-cadherin interface.

Data S3. Alignment of an expanded representative group of integrin I domains used to identify candidate I domain-like proteins and for ancestral sequence reconstruction presented in Figure 4 and Supplemental Figure 10 in FASTA format. Names are presented as NCBI accession numbers, ENSEMBL reference numbers, or ANISEED transcript numbers.

Data S4. Alignment of I domains and related vWFA domains. The alignment used to generate the phylogenetic tree presented in Fig. S9 and Fig. 4. Names are presented as Uniprot identifiers with the region encompassing the domain.

Data S5. Alignment of I domain integrins and their close relatives presented in Fig. 5 and Fig. S11. Only the region encoding the β-propellor following the I domain through the thigh domain was included due to poor alignment outside of this region.
